# Cofactor-Free Serial Amplification of Tau Filaments from Alzheimer’s Disease and Other Tauopathies Depends on the Conformational State of Tau Monomers

**DOI:** 10.1021/jacsau.5c01693

**Published:** 2026-03-11

**Authors:** Zachariah Y. Gabani, Jasdeep Singh, Eric D. Hamlett, Ann-Charlotte Granholm, Martin Margittai

**Affiliations:** † Department of Chemistry and Biochemistry, 2927 University of Denver, Denver, Colorado 80208, United States; ‡ Department of Pathology and Laboratory Medicine, Medical University of South Carolina, Charleston, South Carolina 29425, United States; § Department of Neurosurgery, 129263 University of Colorado Anschutz, Aurora, Colorado 80045, United States

**Keywords:** aggregation, Alzheimer’s disease, amplification assay, amyloid, cofactor, conformation, ensemble, fibril, monomer, Pick’s disease, progressive supranuclear palsy, RT-QuIC, seeding, Tau protein

## Abstract

Tau filaments are a defining characteristic of Alzheimer’s disease (AD) and numerous other neurodegenerative disorders. The deposition of Tau protein into aggregates involves templated recruitment of Tau monomers onto the filament ends via their microtubule-binding repeats. This structural conversion is central to the propagation of Tau pathology, yet its molecular mechanisms are still poorly understood. Specifically, it is unclear whether cofactors are required for templated growth. To gain insights into this process, we probed the serial amplification of pathological Tau filaments from AD, Pick’s disease (PiD), and progressive supranuclear palsy (PSP). These filaments are made from different compositions of three- and four-repeat (3R and 4R) Tau. We observe that AD Tau filaments recruit full-length 3R and 4R Tau in the absence of cofactors at low salt concentration but not at physiological salt concentration and that these filaments can be independently amplified over multiple generations. PiD Tau and PSP Tau filaments can be similarly amplified. The generated filaments retain the cross-seeding properties of the pathological seeds; PSP filaments recruit only 4R Tau, PiD filaments recruit only 3R Tau, and AD filaments recruit both. Regardless of the structural fidelity of the amplification process, we show that the Tau monomer ensemble serves as an entry point for templated growth and that the conformational state of this ensemble (expanded versus compact) determines whether propagation occurs.

## Graphical abstract

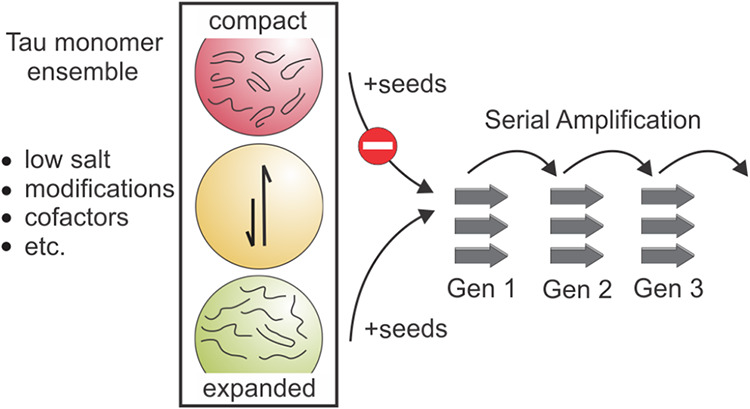

## Introduction

Filaments composed of the microtubule-associated protein Tau are a pathological hallmark of Alzheimer’s disease (AD) and over 20 other neurodegenerative disorders, collectively known as tauopathies.^1–3^

In the adult human brain, six Tau isoforms are expressed due to alternative mRNA splicing from a single gene (*MAPT*) on chromosome 17. These isoforms range in size from 352 to 441 residues and vary in the number of N-terminal inserts (0N, 1N, or 2N) and C-terminal microtubule-binding repeats, with repeat two being either absent or present (3R or 4R). Latter distinction results in the categorization of Tau into three- and four-repeat isoforms. Individual repeats are 31–32 residues in length and span residues 244 to 368 within the protein. Alzheimer’s disease is a mixed tauopathy, in which all Tau isoforms are found in the filaments.^4,5^ In progressive supranuclear palsy (PSP), there is preferential deposition of 4R Tau,^6^ making it a four-repeat tauopathy. In Pick’s disease (PiD), there is preferential deposition of 3R Tau,^7^ making it a three-repeat tauopathy.

Tau monomers are intrinsically disordered,^8,9^ but when assembled into fibrils, the repeat regions form a parallel and in-register β-sheet structure, in which identical residues from neighboring proteins are perfectly stacked on top of each other.^10,11^ Although these early insights were gained from fibrils that were formed *in vitro*, pathological Tau filaments proved to share an in-register arrangement of β-strands. The cryo-electron microscopy analysis of Tau filaments isolated from AD brain provided the first high-resolution structure.^12^ The filaments, which are predominantly of the paired helical filament type, were observed to have a cross-β/β-helical fold that encompasses residues 304/306–378/380 in repeats 3 and 4 and the immediately adjacent C-terminal region.^12,13^ These residues are common to all Tau isoforms and provide an explanation for their joint incorporation into the filament.^12^ Residues outside the structured region form a fuzzy, disordered coat that surrounds the central core.^14,15^ Filaments in PiD and PSP were found to have β-sheet folds that differ from the one in AD involving residues 254–378 ^16^ and 272–381,^17^ respectively. Structural evidence from other diseases corroborated that each tauopathy is characterized by a specific fold.

The pathology of Tau spreads transsynaptically from one neuron to another,^18,19^ as well as from neurons to other cell types such as oligodendrocytes^20^ and spatiotemporally via defined pathways throughout the brain,^21–23^ suggesting that small Tau aggregates serve as seeds that recruit naïve Tau monomers onto their ends. This structural conversion has been observed in multiple *in vivo* model systems^24,25^ and together with the observation of specific Tau folds indicates that the protein shares key properties with prions.^26^ However, the molecular mechanisms of seeded or templated Tau aggregation remain poorly resolved. It is unknown how different environmental conditions modulate the recruitment of Tau onto the fibril end.

Early on it was shown that negatively charged cofactors greatly enhance the spontaneous aggregation of Tau protein *in vitro*.^27–30^ The use of the cofactor heparin also facilitated the amplification of minute quantities of pathological fibrils from AD and other tauopathies.^31–35^ These assays mechanically fracture Tau fibrils into smaller seeds to then recruit either full-length^31^ or truncated monomers^32–35^ into the fibrils. More recently, sarkosyl-insoluble material from AD brain was successfully used to amplify full-length Tau in the absence of cofactors.^36^ These Generation 1 fibrils possessed the paired helical filament structure of the original fibrils, but surprisingly they lacked the ability to initiate seed amplification of another generation.^37^ This suggested that cofactors or other components are needed to sustain pathological fibril growth. In support of this, RNA and heparan sulfate are found in AD-filament containing lesions in AD.^38,39^ Furthermore, cofactors were necessary for templated aggregation of synthetic Tau fibrils.^40,41^ Additionally, fibrils that were generated on a platform in which heparin was immobilized to the surface, required cofactor for further seeding, although the fibrils had no cofactor incorporated.^42^ Contrasting these findings, Lövestam et al.^43^ demonstrated that the core region of Tau is sufficient to form paired helical filaments without the addition of cofactors. Even more, it was shown that filaments composed of the core region were able to recruit full-length Tau.^44^ These seemingly contradictory findings raise the question of what determines whether full-length Tau monomers can or cannot be recruited into the fibril. Here we set out to address this question. We show that filaments from AD, PSP, and PiD can be serially amplified in the absence of cofactors and in low salt buffer but that physiological salt concentrations are inhibitory. The findings demonstrate that Tau protein alone encodes all of the information for fibril propagation and show that the conformational status of the Tau monomer ensemble dictates whether templated growth proceeds.

## Results

### Alzheimer’s Disease Brain Tissue Homogenates Seed Both 3R and 4R Tau Isoforms

To assess the seeding competency of Tau fibril-containing brain samples, tissue extracts from the frontal cortex of three separate AD subjects, AD 1–3, and three nondemented controls (Table S1) were mixed with recombinant full-length 3R Tau protein (0N3R) in the presence of a low salt phosphate buffer (10 mM sodium phosphate, pH 7.4) and excess reducing agent (10 mM dithiothreitol). These buffer conditions (and others) were successful in generating paired helical filaments from truncated Tau.^43^ The seeding reactions contained more than a 10-fold excess of recombinant 3R Tau relative to total brain protein (see Materials and Methods section). The number of actual pathological fibril ends in these mixtures, compared to soluble Tau monomers available for recruitment, is miniscule. To accelerate the seeded aggregation, we employed a modified Real-Time Quaking-Induced Conversion assay or short RT-QuIC^45^ in which the samples were periodically shaken and quiescently incubated in a microplate to promote fibril fracture and growth. Aggregation was monitored after the addition of Thioflavin T (ThT), a dye that exhibits increased fluorescence upon binding to the emerging β-sheet-rich fibrils.^46^

Employing this assay, we observed that 3R Tau monomers aggregated in the presence of all three AD brain extracts but not in the presence of the controls (Figure 1a). This suggested that the pathological seeds present in the AD samples, but not in the controls, were able to recruit 3R Tau monomers. These aggregates are referred to as Generation 1. To validate this finding and to quantify the degree of aggregation, we next sedimented all completed reactions using ultracentrifugation. The supernatants and pellets were analyzed by SDS-PAGE and Coomassie staining (Figure S1a,b). Quantification revealed that for AD-templated reactions, more than 90% of 3R Tau was distributed in the aggregate-containing pellet, whereas in reactions with extracts from control brains, the protein remained predominantly (∼95%) in the soluble supernatant (Figures 1b and S1a,b).

**Figure 1:**
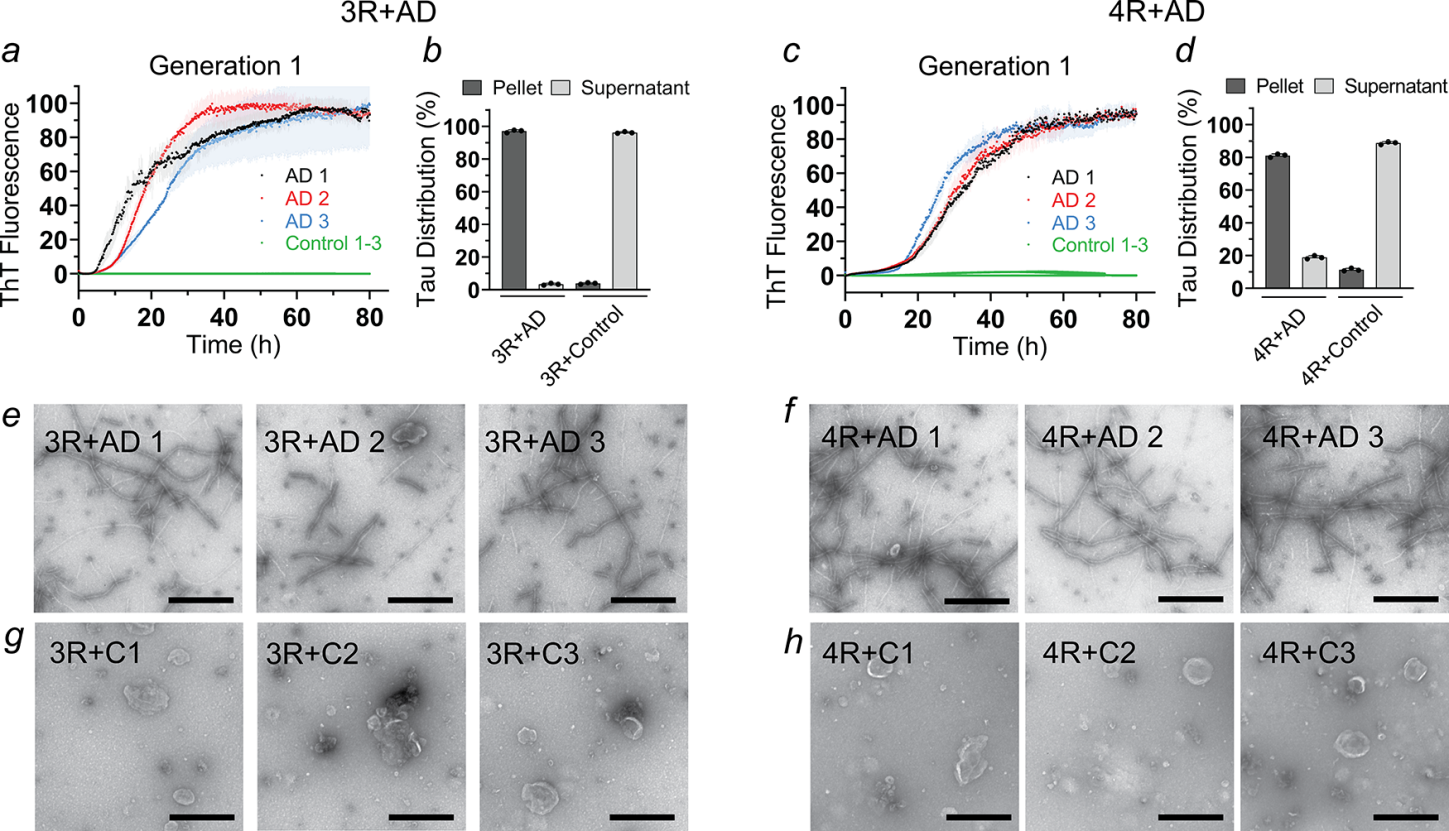
AD brain homogenates convert full-length 3R and 4R Tau monomers into aggregates in the absence of cofactors. Recombinant Tau monomers (10 μM) were mixed with AD (AD 1–3) or control (C1–3) brain homogenates (30 μg/mL) in 10 mM sodium phosphate buffer (pH 7.4) and incubated at 37 °C using the standard RT-QuIC protocol. This resulted in the formation of Generation 1 fibrils. ThT traces and sedimentation analyses of reactions with 3R Tau (a, b) and 4R Tau monomers (c, d), respectively. The SDS-PAGE gels underlying the sedimentation analyses are shown in Figure S1. Each biological replicate was repeated in triplicate (*n* = 3). Error bars represent means ± SD. Representative negative stain transmission electron micrographs of 3R Tau + AD 1–3 (e), 4R Tau + AD 1–3 (f), 3R Tau + C1–3 (g), and 4R Tau + C1–3 (h). All images were taken after completion of the seeding reactions. Scale bars, 500 nm.

Next, we tested whether the AD brain extracts could also template the growth of full-length 4R Tau (2N4R) under the same low salt reaction conditions. 4R Tau incubated with AD 1–3 or control brain extracts showed ThT growth characteristics similar to those with 3R Tau (Figure 1c). Sedimentation and densitometric analyses corroborated the ThT outcomes; 4R Tau aggregated in AD-templated reactions (∼80%) while remaining largely soluble (∼90%) in the presence of control brain extracts (Figures 1d and S1c,d). The data suggest that seeds present in the AD brain extracts recruit not only 3R Tau, but also 4R Tau, as expected for these fibrils.^12^ Spontaneous aggregation of the monomers can be excluded since the protein remained in the supernatant when extracts from control brains were used. Similarly, when monomers were incubated on their own, in the absence of any extracts, no aggregation was observed (Figure S2), confirming that seeds are necessary for Tau monomers to aggregate under the given low salt buffer conditions. To exclude the possibility that ThT facilitated the aggregation from AD brain extracts, the templating experiments were repeated in the absence of ThT. As before, the majority of 3R and 4R Tau (>80%) were found in the aggregate-containing pellets (Figure S3). Interestingly, when the salt concentration of the reaction was increased by adding 150 mM NaCl, neither 3R nor 4R Tau monomers aggregated in the presence of the AD 1–3 brain extracts (Figure S4), indicating that the low salt conditions are important for facilitating seeded growth. Markedly, once the fibrils were formed, the addition of salt did not cause any disaggregation, suggesting that fibril stability was preserved (Figure S5). Salt addition, however, had measurable effects on the conformational ensembles of Tau monomers as the hydrodynamic radii decreased from 9.2 to 6.8 nm for 3R Tau and from 10.2 to 7.1 nm for 4R Tau (Figure S6). Lastly, when oxidized 3R or 4R Tau proteins (with inter and intramolecular disulfide bonds between their native cysteines, respectively) were added to the AD brain extracts (in the absence of reducing agent) under the original low salt conditions, no aggregation was observed (Figure S7), indicating that oxidized 3R and 4R Tau proteins do not incorporate into AD seeds and that a reducing environment is necessary for successful seeding.

To visualize and confirm the presence of fibrils, we next imaged the end products from our amplification reactions (Figure 1a,c) using negative stain electron transmission microscopy (TEM). All samples that were seeded with AD extracts contained fibrils (Figure 1e,f), whereas samples that were seeded with control extracts did not (Figure 1g,h). The combined data demonstrate that AD-derived seeds can be efficiently amplified with wild-type full-length 3R and 4R Tau monomers without the addition of cofactors but that the reactions require specific buffer conditions to proceed.

### Cofactors Are Not Required for the Serial Amplification of AD Fibrils

While we were successful in templating and amplifying AD Tau with recombinant 3R and 4R Tau monomers (Generation 1 or Gen 1 for short) in the absence of cofactors, we were interested in assessing whether the fibrils from Generation 1 could initiate another round of amplification and whether this process could be serially repeated. Our rationale for performing a multigeneration/serial amplification assay was that if fibrils (Generation 1) could be formed in the absence of cofactor, then, along or over successive generations, these fibrils should be sufficient for stable propagation of Tau protein aggregates. To investigate this, we conducted systematic serial amplification steps referred to as Generation 2–6, where 1% of the end products from a previous generation were used to seed/template 3R or 4R Tau monomers in the next generation. As an example, for setting up Generation 2 reactions, we sonicated the reaction end products from Generation 1 samples and incubated them with new recombinant 3R or 4R Tau monomers at a 1% seed-to-monomer ratio. Thus, each generation involved a 100-fold dilution of the original brain extract (which had a total protein concentration of 30 μg/mL in Generation 1), resulting in an overall dilution to 1 × 10^–10^ by Generation 6. To visualize the seeds that were used for initiating the serial reactions, sonicated fibrils from Generation 1 were inspected by TEM. The images confirmed the presence of short fibrillar 3R or 4R Tau seeds with an average length ranging from 63 to 68 nm (Figure S8).

Next, 3R Tau seeds from Generation 1 were incubated with recombinant 3R Tau monomers using the same RT-QuIC reaction conditions that were used for the generation of the initial set of fibrils. Notably, the 3R Tau seeds from Generation 1 successfully templated 3R Tau monomers, as evident from the ThT growth curves (Figure 2a, upper panel) and the sedimentation-densitometric analyses showing ∼90% of protein in pellet fractions (Figures 2b, and S9, upper panels). Importantly, across multiple generations (monitored here up to Generation 6), 3R Tau seeds efficiently recruited 3R Tau monomers, converting them into fully aggregated states without the need for added cofactors (Figures 2a,b and S9, lower panels). Similar results were obtained for the serial propagation of the 4R Tau (Generations 2–6). The ThT kinetics and sedimentation analyses indicated efficient conversion of soluble 4R Tau monomers to aggregated states in the presence of fibril seeds from the previous generation, without assistance of external cofactors (Figures 2c,d and S10). Notably, at elevated salt, serial amplification failed. Neither 3R nor 4R Tau monomers were recruited onto Generation 1 seeds when 150 mM NaCl was added (Figure S11), in agreement with the seeding properties of the original AD extracts (Figure S4). Even when the seed concentration was increased from 1 to 10%, Tau monomers failed to be recruited (Figure S11), suggesting that seed concentration is not the limiting factor, but that it is the altered conformational state of the monomer ensemble (Figure S6) that prohibits serial amplification at physiological salt. TEM analyses of samples from the last amplified generation (Generation 6 of both 3R and 4R Tau) at low salt revealed that Tau aggregates maintained their fibrillar structure (Figure S12).

**Figure 2:**
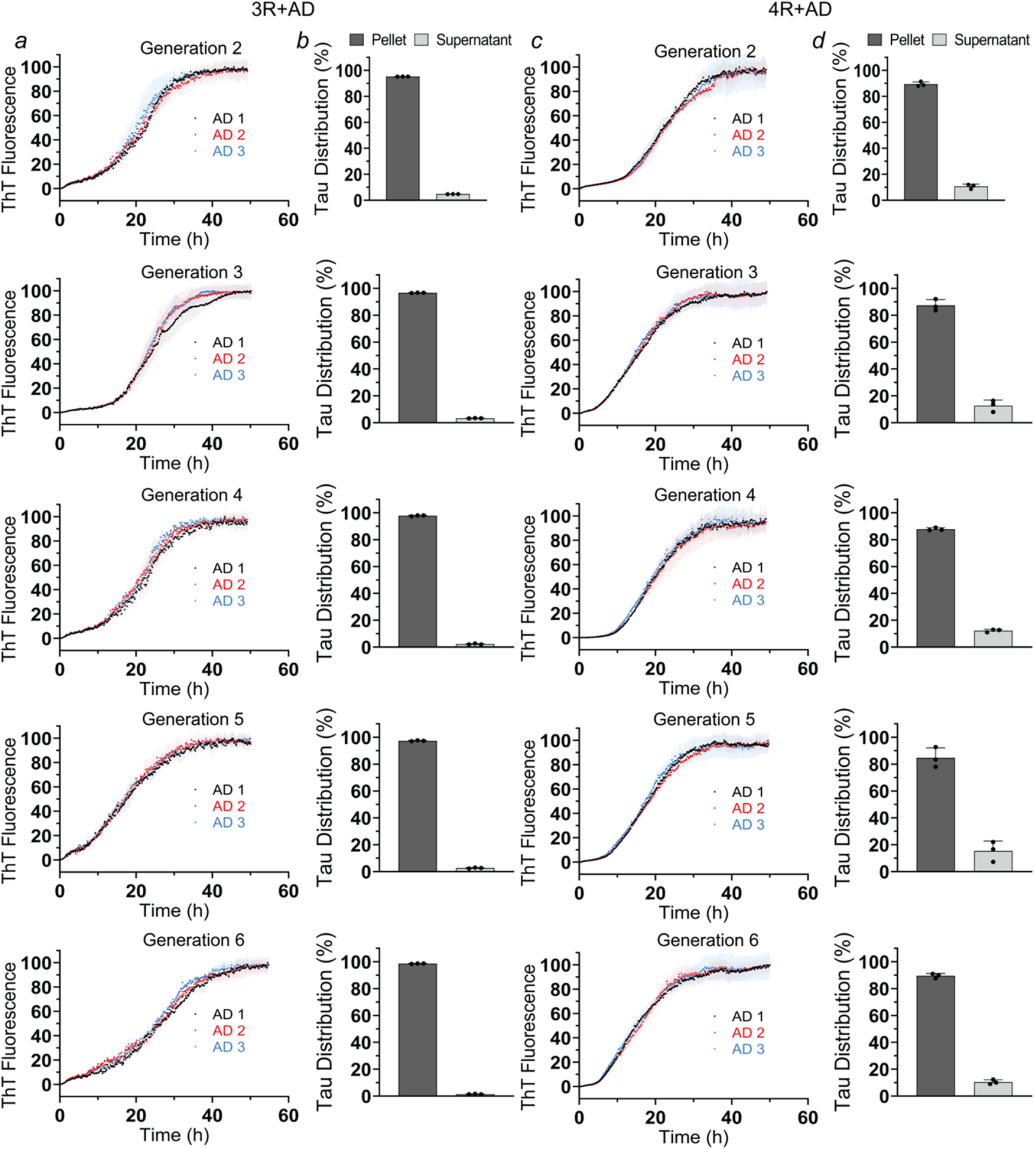
Cofactors are not required to serially amplify AD fibrils. Recombinant Tau monomers (10 μM) were mixed with sonicated AD fibrils (Generation 1) in 10 mM sodium phosphate buffer (pH 7.4) at a 1:100 seed-to-monomer ratio and incubated at 37 °C using the standard RT-QuIC protocol. This resulted in the formation of Generation 2 fibrils. ThT traces and sedimentation analyses for reactions that included 3R Tau (a, b) and 4R Tau monomers (c, d) are presented in the top panels. The amplification steps were repeated, leading to the formation of fibril generations 3–6 (lower panels) in which the original brain material was successively diluted (1:100 for each generation). The SDS-PAGE gels underlying the sedimentation analyses for seeded reactions with 3R Tau and 4R Tau monomers are shown in Figures S9 and S10, respectively. Each biological replicate was repeated in triplicate (*n* = 3). Error bars represent means ± SD. AD 1–3 reflect reactions with seeds from the immediately preceding fibril generation. These seeds descended from the original brain homogenates.

Given that AD brain extracts seed both 3R and 4R Tau monomers (Figure 1), we next asked whether serial amplification of 3R or 4R Tau affected the ability to cross-seed. To test this, 3R and 4R Tau fibrils obtained from Generation 6 were sonicated and incubated with monomers of the other isoform. 4R Tau monomers were mixed with 3R Tau seeds and vice versa. The temporal increase in ThT fluorescence and sedimentation analysis revealed that 4R Tau monomers were fully recruited onto 3R Tau seeds (Figures 3a,b and S13a); conversely, 3R Tau monomers were fully recruited onto 4R Tau seeds (Figures 3c,d and S13b). Although there are differences in the kinetics between the two reactions, the results indicate that the hallmark cross-seeding abilities of the original AD fibrils were preserved.

**Figure 3:**
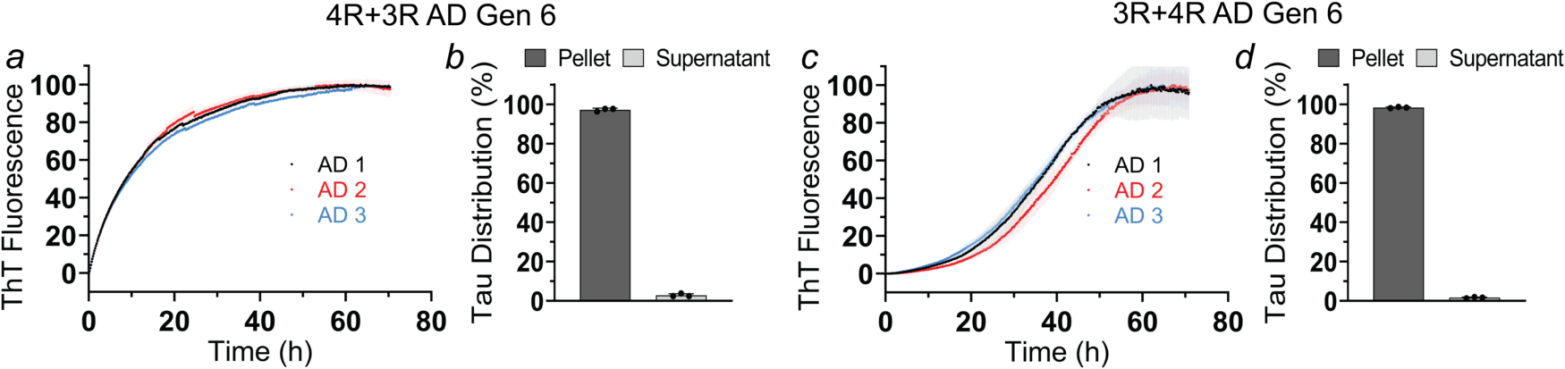
Cross-seeding abilities of AD fibrils are preserved after serial amplification. Recombinant Tau monomers (10 μM) were mixed with AD 1–3 seeds (Generation 6) composed of the other isoform (i.e., 3R Tau seeds for 4R Tau monomers and 4R Tau seeds for 3R Tau monomers) at a 1:100 seed-to-monomer ratio and incubated at 37 °C using the RT-QuIC protocol. ThT traces and sedimentation analyses of 4R Tau monomers grown onto 3R Tau seeds (a, b) and 3R Tau monomers grown onto 4R Tau seeds (c, d). The SDS-PAGE gels underlying the sedimentation analyses are shown in Figure S13. Each biological replicate was repeated in triplicate (*n* = 3). Error bars represent means ± SD.

### AD-Amplified Fibrils Induce Intracellular Tau Aggregation

Tau aggregation in human embryonic kidney 293 (HEK293) cells has been widely used to assess the seeding propensity of exogenously introduced fibrils.^47–50^ We next sought to evaluate the seeding activity of our fibrils that were generated through serial amplifications. Specifically, 3R and 4R Tau seeds (obtained through sonication of reaction end products) from Generation 1 and Generation 6 fibrils were transfected into HEK293 cells expressing the P301S variant of 4R Tau (2N4R) tagged with a yellow fluorescent protein at its C terminus.^49^ Cells treated with 10 mM phosphate buffer alone served as controls. After incubation for 24 h at 37 °C, cells were imaged and analyzed for intracellular puncta, which serve as a readout for seeded aggregation. We observed that all externally offered seeds, regardless of their composition or generation (1 versus 6), led to the formation of puncta (Figures 4a,b and S14), whereas buffer controls did not. Quantification revealed that the number of puncta was similar for all seeds (Figure 4d and Table S2). The results suggest that Tau fibrils, serially amplified from AD extracts in the absence of cofactors, can recruit Tau monomers inside the cell.

**Figure 4:**
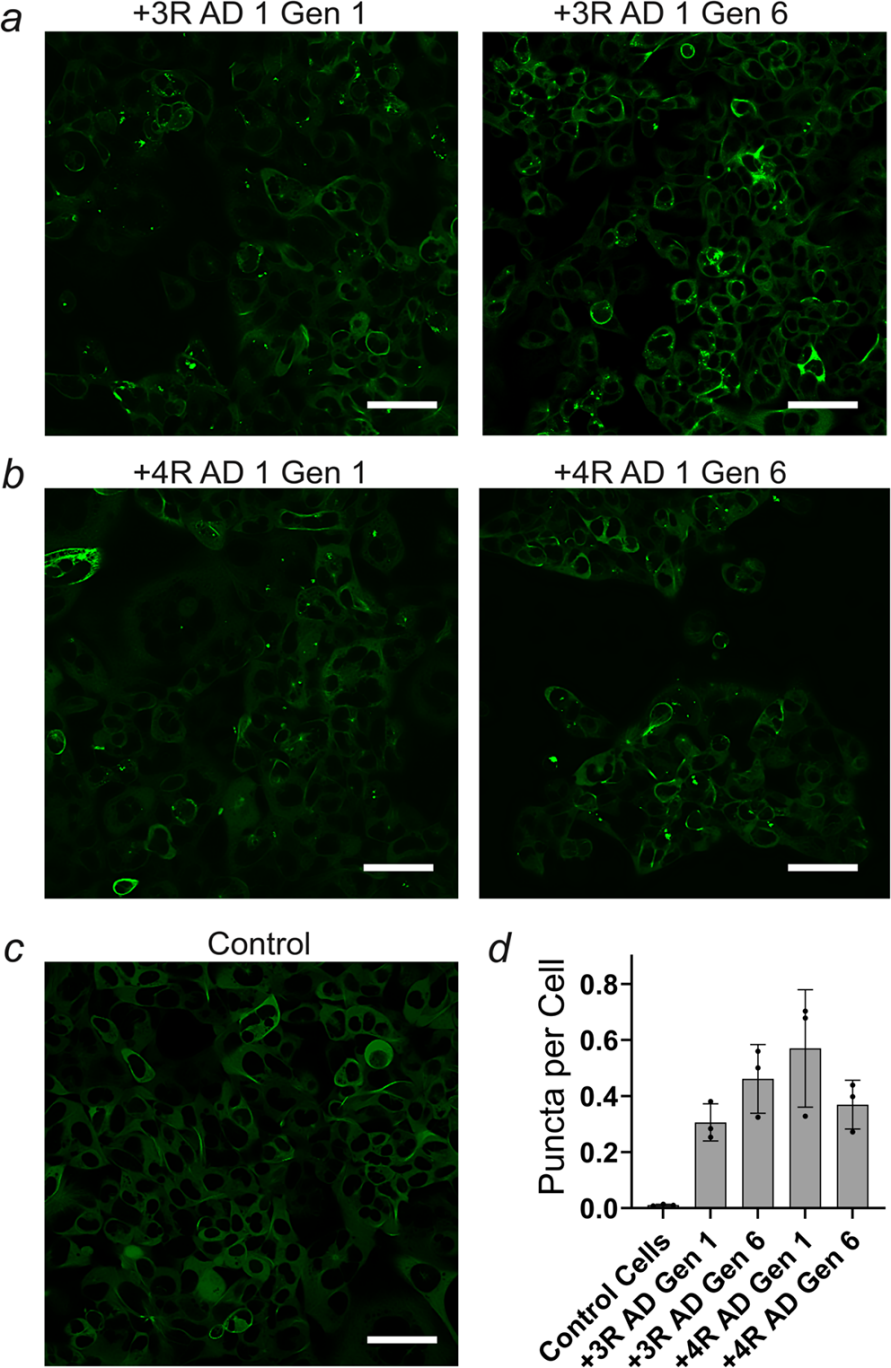
Tau fibrils amplified from AD brain homogenates induce intracellular Tau aggregation. Monoclonal HEK293 cells that expressed htau40P301S (a variant of 2N4R Tau) tagged with EYFP at the C terminus were transfected with Tau seeds and incubated for 24 h at 37 °C. Representative images of cells transfected with AD 1 seeds amplified with 3R Tau monomers (a) or 4R Tau monomers (b). Generation 1 (left panels). Generation 6 (right panels). Cells transfected in the absence of seeds served as a control (c). Scale bars, 40 μm. The number of puncta per cell was counted for each biological replicate (AD 1–3) (d). Error bars represent means ± SD. Cell images for biological replicates (AD 2, AD 3) and additional quantifications are provided in Figure S14 and Table S2, respectively.

### Cofactors Are Not Required for the Amplification of Tau Filaments from PiD and PSP

The paired helical filament fold in AD accommodates all Tau isoforms. Next, we wanted to determine whether Tau filaments in PiD and PSP, which possess folds that are made exclusively of 3R Tau (PiD) or 4R Tau (PSP), could also be amplified from their respective brain homogenates. For this purpose, we followed the same low-salt RT-QuIC protocol that we used for amplifying AD filaments (Figure 1). First, brain extracts from PiD and PSP (Table S1) were mixed with full-length 3R or 4R Tau monomers, respectively, and then the mixtures were incubated with intermittent shaking. The ThT traces for PiD samples exhibited time-dependent increases in fluorescence, indicative of fibril formation (Figure 5a). This finding was confirmed by sedimentation analysis of the reaction end products, demonstrating that the majority of recombinant 3R Tau protein was distributed into the pellet (Figures 5b and S15a). Similar results were obtained for the PSP samples: a time-dependent increase in ThT fluorescence (Figure 5c) and a prominent 4R Tau protein distribution into the pellet (Figures 5d and S15b). The addition of 150 mM NaCl to the reactions effectively blocked Tau amplification (Figure S16), suggesting that the increased salt concentration in the samples was inhibitory, as it was in amplifications with AD extracts (Figure S4). Note that 3R and 4R Tau monomers incubated with homogenates from control brains did not aggregate under the low salt conditions (Figure 1), indicating that the reactions at low salt are templated and not spontaneous. When the samples were analyzed by TEM, long filaments were observed for both PiD- (Figure 5e) and PSP-seeded (Figure 5f) reactions, validating that the aggregates were not amorphous. The combined data suggest that brain extracts from PiD and PSP can seed full-length 3R or 4R Tau monomers without the addition of cofactors.

**Figure 5:**
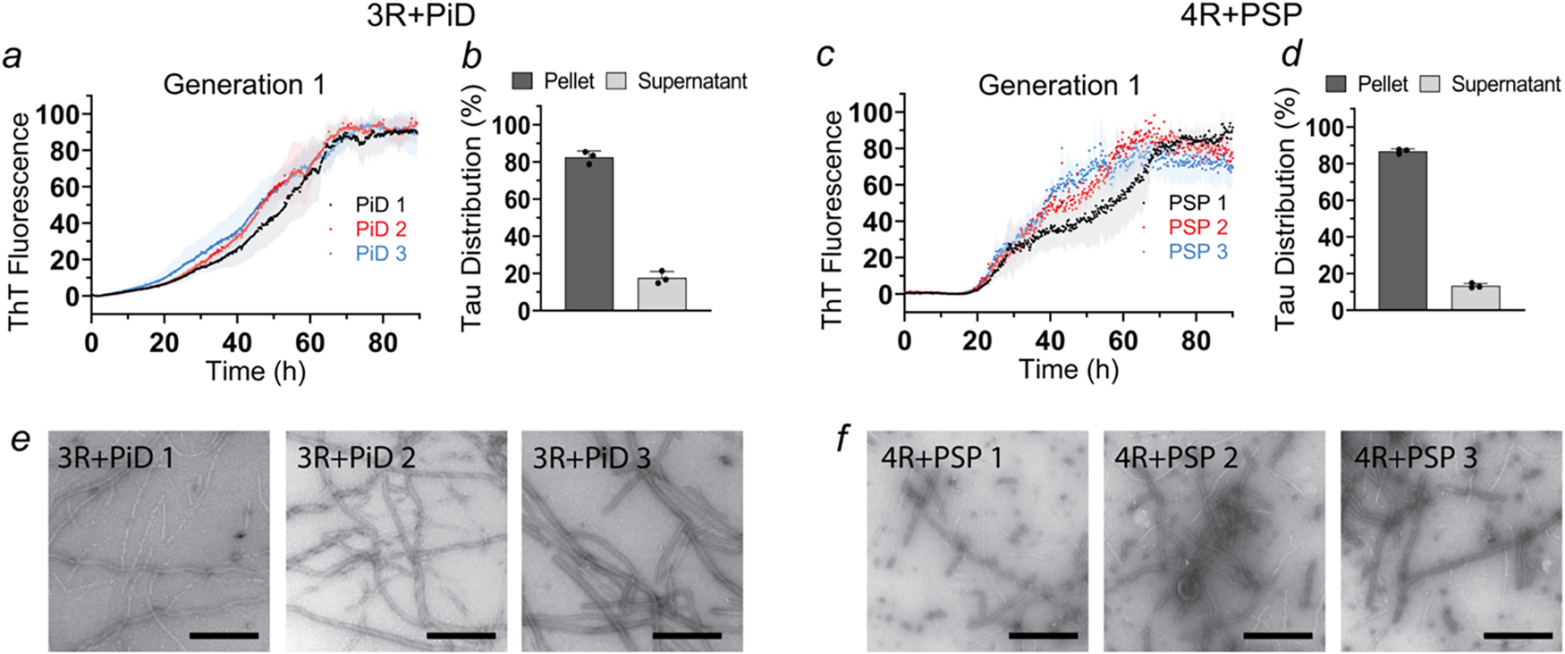
Cofactors are not required to amplify Tau filaments from PiD and PSP brain homogenates. Recombinant Tau monomers (10 μM) were mixed with PiD (PiD 1–3) or PSP brain (PSP 1–3) homogenates (30 μg/mL) in 10 mM sodium phosphate buffer (pH 7.4) and incubated at 37 °C using the RT-QuIC protocol. This resulted in the formation of Generation 1 fibrils. ThT traces and sedimentation analyses of PiD-seeded reactions with 3R Tau (a, b) and PSP-seeded reactions with 4R Tau monomers (c, d), respectively. The SDS-PAGE gels underlying the sedimentation analyses are shown in Figure S15. Each biological replicate was repeated in triplicate (*n* = 3). Error bars represent means ± SD. Representative negative stain transmission electron micrographs of 3R Tau + PiD 1–3 (e) and 4R Tau + PSP 1–3 (f). All images were taken after completion of the seeding reactions. Scale bars, 500 nm.

Next, we were interested in determining what the cross-seeding properties of these Generation 1 fibrils are and whether they could be amplified further. For this purpose, the fibrils were first sonicated, generating small seeds with an average length of ∼67 nm (Figure S17). The seeds were then mixed with either 3R or 4R Tau monomers and subjected to the RT-QuIC assay. The ThT traces for the PiD samples revealed that 3R Tau monomers grew onto Generation 1 seeds, whereas 4R Tau monomers did not (Figure 6a). These findings were confirmed by sedimentation analysis that showed all 3R Tau protein to be in the pellet and all 4R Tau protein to be in the supernatant (Figures 6b and S18a,b). The ThT traces for the PSP samples exhibited characteristics opposite to those observed for PiD. 4R Tau monomers grew onto Generation 1 seeds, while 3R Tau monomers did not (Figure 6c). These findings were further corroborated by sedimentation analysis, which indicated that all 4R Tau protein was distributed into the pellet while all 3R Tau protein remained in the supernatant (Figures 6d and S18c,d). TEM images confirmed the presence of fibrils in PiD-seeded reactions that included 3R Tau monomers (Figure 6e) and PSP-seeded reactions that included 4R Tau monomers (Figure 6f). Both reactions are homotypic, meaning that monomers and seeds are made of the same isoform. Heterotypic reactions, those in which there is a mismatch in isoforms between seeds and monomers, produced no fibrils (Figure 6g,h). These data agree with the isoform-specific composition of Tau fibrils found in PiD and PSP^16,17^ and highlight the ability to serially amplify the original disease aggregates in an *in vitro* environment using recombinant full-length Tau proteins.

**Figure 6:**
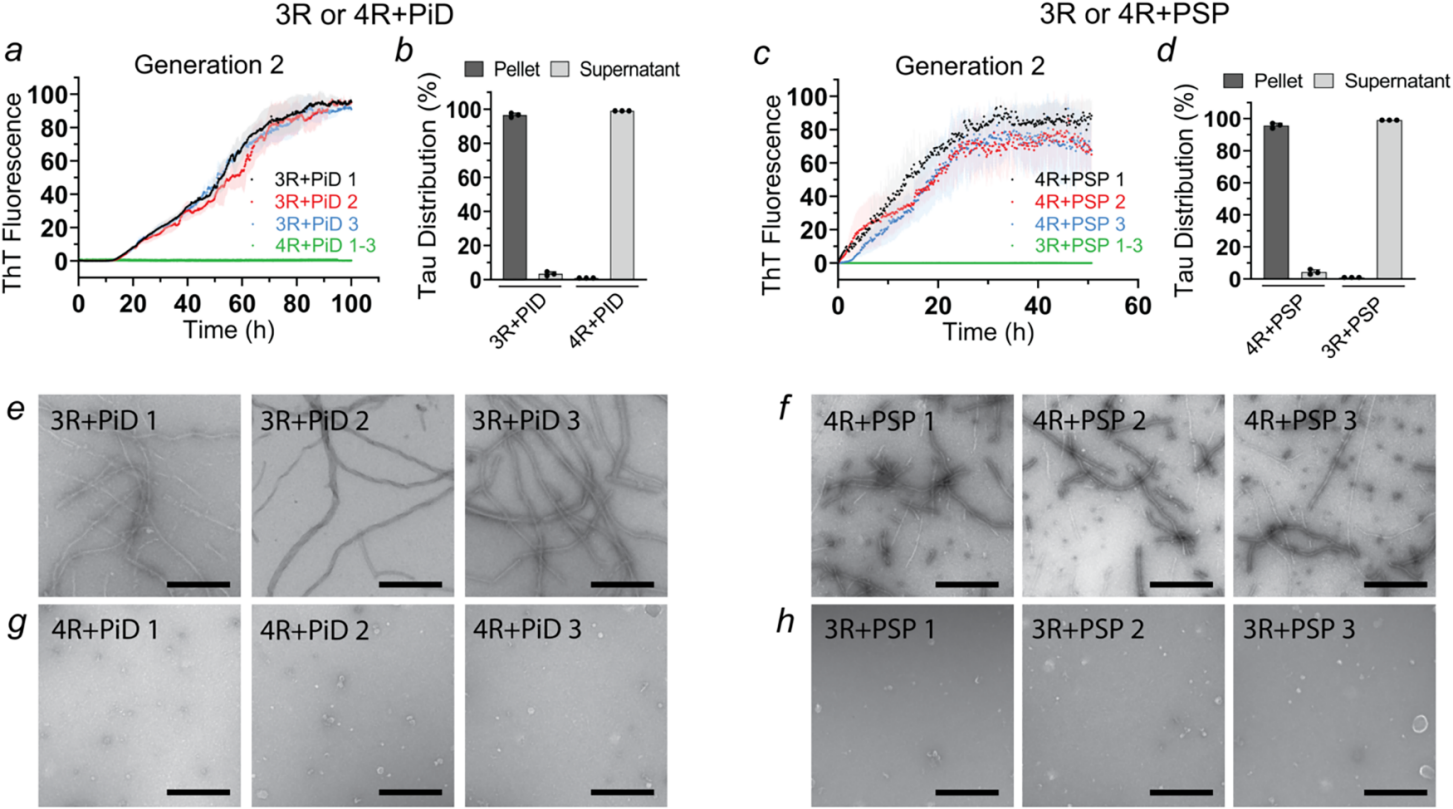
Generation 1 Tau fibrils from PiD and PSP can be serially amplified while retaining their characteristic cross-seeding properties. Recombinant Tau monomers (10 μM) were mixed with sonicated fibrils from PiD or PSP (Generation 1) in 10 mM sodium phosphate buffer (pH 7.4) at a 1:100 seed-to-monomer ratio and incubated at 37 °C using the standard RT-QuIC protocol. For homotypic seeding, this resulted in the formation of Generation 2 fibrils. For heterotypic seeding, there was a robust barrier. ThT traces and sedimentation analyses for PiD-seeded reactions (a, b) and PSP-seeded reactions (c, d). The SDS-PAGE gels underlying the sedimentation analyses for Generation 1 seeded reactions are shown in Figure S18. Each biological replicate was repeated in triplicate (*n* = 3). Error bars represent means ± SD. Representative negative stain transmission electron micrographs of 3R Tau + PiD 1–3 (e), 4R Tau + PSP 1–3 (f), 4R Tau + PiD 1–3 (g), and 3R Tau + PSP 1–3 (h). All images were taken after completion of the seeding reactions. Scale bars, 500 nm. In all cases, PiD 1–3 and PSP 1–3 reflect reactions with seeds from Generation 1 fibrils. These seeds descended from aggregates in the original brain homogenates.

## Discussion

The recruitment of Tau monomers onto the fibril end is a central step in the propagation of Tau filaments in AD and other neurodegenerative disorders. Whether this step requires the presence of negatively charged cofactors remains unresolved. Here, we set out to address this question using AD brain extracts as a source of seeds and recombinant wild-type Tau as a substrate. We observed that both 3R Tau and 4R Tau robustly elongated onto AD seeds, leading to a first generation of *in vitro*-formed filaments. These findings are in agreement with previous observations that demonstrated cell-free amplification of AD filaments.^36,37^ To test whether these Generation 1 aggregates could propagate further, the filaments were subjected to serial amplification in which the original total protein concentration from the brain was successively diluted over 100-billion-fold relative to the concentration of recombinant Tau. At this dilution, the concentration of brain-derived cofactors is negligible. We observed that Tau aggregates could be successfully amplified by using both 3R and 4R Tau. Importantly, the amplified filaments retained the cross-seeding properties of AD filaments, where 3R Tau could elongate onto 4R Tau filaments, and vice versa. The filaments also exhibited full seeding competency when transfected into HEK293 cells overexpressing the full-length Tau protein harboring the P301S mutation. In an extension to these studies, we observed that filaments from PSP and PiD could also be serially amplified in the absence of cofactors and that the filaments showed their characteristic seeding behavior, with PSP-seeded filaments only recruiting 4R Tau and PiD-seeded filaments only recruiting 3R Tau. These properties are consistent with the previously observed seeding barriers of brain-derived filaments from PiD^16,35^ and PSP.^32^ The ability to serially amplify pathological Tau filaments with recombinant wild-type Tau protein in the absence of cofactors is shown for the first time. However, the question arises why cofactors were required for sustaining seeded aggregation in some studies but not here.

One explanation could lie in the fibril structure. For example, when Tau fibrils are formed spontaneously *in vitro* in the presence of cofactors such as heparin^41^ or RNA,^40,41^ the cofactors can exert selective pressures that favor specific fibril folds. These folds require cofactors for fibril stability and templated growth.^40,41^ Cryo-EM data revealed that the structures of Tau filaments formed in the presence of heparin^51^ and RNA^52^ varied significantly from those observed in AD and other tauopathies. On the contrary, peptide fragments encompassing the core region of AD filaments were sufficient to form paired helical filaments in the absence of cofactors,^43^ suggesting that these factors are not needed to stabilize the AD fibril fold and to facilitate seeding.

Although Tau is an intrinsically disordered protein, early evidence indicated that its structure is significantly different from that of a random coil. It was observed that the N- and C-terminal regions can fold back onto the repeat region like a paperclip forming a more closed compact conformational state.^53^ Cofactors such as heparin are able to release long-range interactions between the two termini and the microtubule-binding repeats, leading to a globally expanded conformation with enhanced aggregation propensity.^54^ Importantly, we previously observed that these two conformational states (compact versus expanded) exist even in the absence of cofactors,^55,56^ suggesting that aggregation of full-length Tau does not necessitate the presence of heparin or RNA. Changes in the conditions can shift the distributions of these states, thereby having a direct impact on aggregation. In the present work, we showed that a switch from physiological salt to low salt conditions resulted in an increased hydrodynamic radius of 3R and 4R Tau monomers. These data are consistent with small-angle X-ray scattering experiments that indicated a conformational expansion of full-length Tau monomers under low salt.^57^ This conformational shift can make Tau more prone to aggregation. Congruent with this view, we observed that seeded aggregation proceeded at low salt, but not at physiological concentrations, regardless of whether seeds were from AD, PiD, or PSP, suggesting that the conformational state of the monomer ensemble and not the structure of the seeds determines whether propagation occurs. Additional support for this interpretation comes from work by Chakraborty et al., who achieved spontaneous aggregation of full-length Tau (2N4R) without cofactors under low salt conditions.^58^ Markedly, the generated filaments (without altering conditions) possessed the ability to seed.^58^ It is likely that other parameters, such as pH and types of buffers and salts, affect the monomer ensemble as well. Additionally, phosphorylation at specific sites within Tau can cause a more expanded conformation,^59,60^ providing another path for aggregation. Furthermore, it has been demonstrated that 12 phosphomimetic mutations trigger Tau to form paired helical filaments and that the variant can be recruited into pathological filaments via seeding.^61^ There is also ample evidence that specific disease mutations in Tau cause a local opening of the monomer conformation.^62–65^ These observations highlight the existence of alternate ways for Tau to undergo a transition into expanded states. The combined evidence suggests that the open conformation is a prerequisite for nucleation and for sustaining growth onto pathological seeds.

Recent findings indicate that effective collisions of monomers with the fibril end are the bottleneck in fibril elongation of PI3K-SH3, a protein without substantial residual structure.^66^ Although the molecular steps in Tau elongation are still poorly understood, a compact conformation of Tau could lower the number of successful monomer fibril end collisions, as the monomer would have to transition into an open state. Notably, in compact Tau, the aggregation-prone hexapeptide motifs (^275^VQIINK^280^ and ^306^VQIVYK^311^) in the microtubule-binding repeats are shielded by local structure.^64,67^ Opening the protein and exposing these hydrophobic residues could facilitate critical interactions with the fibril end. An intramolecular disulfide bond between the two native cysteines in 4R Tau results in a different type of compact monomer in which the second and third microtubule-binding repeats are covalently linked.^68^ In the current study, we observed that this monomer is unable to grow onto AD seeds. A similar barrier was also observed for disulfide-linked dimers of 3R Tau. These findings provide further support for the conclusion that Tau monomers need to be in an open and accessible conformation to successfully engage with the fibril end.

Although we observe that Tau filaments can be propagated over multiple generations, and the fibrils preserve their general seeding characteristics, this does not exclude the possibility that there could be conformational adaptations. Indeed, AD filaments that were used for seeding in SH-SY5Y cells showed small structural changes when amplified in these cells.^69^ The molecular structures of the fibrils generated herein will have to be determined in the future. Regardless, our findings demonstrate that Tau protein alone (in the absence of a cofactor) is sufficient to propagate onto pathological seeds. This study sheds new light on the monomer ensemble as an entry point for templated growth. The conformational status of this ensemble (compact vs expanded) is a critical determinant for fiber propagation. The fidelity of propagation could be governed by another set of rules. Understanding how cofactors, salts, modifications, and other biomolecules modulate these two aspects of aggregation (monomer ensembles and elongation fidelity) should provide exquisite control over the assembly process. This knowledge should be of benefit for generating unlimited quantities of pathological fibrils *in vitro* and in developing new therapeutic strategies that interfere with Tau propagation and slow disease progression.

## Materials and Methods

### DNA Constructs

Gene-optimized DNA inserts encoding human wild-type 0N3R Tau and 2N4R Tau were synthesized and cloned into pET-28 expression vectors by Biomatik (Ontario, Canada). The genes were inserted using the Nco1 and Xho1 restriction sites, with two stop codons preceding the Xho1 site to eliminate all tags.

### Expression and Purification

0N3R (3R Tau) and 2N4R (4R Tau) were expressed and purified by adapting our previously published protocols.^11,70^ Specifically, BL21 (DE3)-competent *Escherichia coli* cells were transformed with the respective pET-28 vectors by heat shock and plated on LB agar containing 50 μg/mL kanamycin. A single colony was picked and grown in LB medium (20 μg/mL kanamycin) at 37 °C while being shaken at 200 rpm for 16 h. The culture was diluted 1:100 into fresh LB medium (20 μg/mL kanamycin) and incubated at 37 °C and 200 rpm until the optical density at 600 nm reached 0.7–1.0. Protein expression was induced by adding 0.5 mM isopropyl β-D-1-thiogalactopyranoside (IPTG), followed by incubation at 37 °C while being shaken at 200 rpm for 3 h. Cells were harvested by centrifugation and resuspended in buffer containing 1 mM EDTA, 50 mM β-mercaptoethanol, 500 mM NaCl, and 20 mM Pipes, pH 6.5. For protein extraction, cells were heated at 80 °C for 15 min, followed by tip sonication on ice for 1 min at 50% amplitude. Soluble Tau was separated from insoluble material by centrifugation at 15,000*g* for 30 min. Proteins were precipitated with 55–60% (w/v) ammonium sulfate while mixing for 16 h at 22 °C. Precipitated protein was collected by centrifugation at 20,000*g* for 10 min and resolubilized in 2 mM DTT. The solution was tip-sonicated on ice for 4 min, filtered through a 0.45 μm GxF/GHP syringe filter, and loaded onto a Mono S 10/100 GL column (GE Healthcare). Elution was performed with a linear NaCl gradient (50–1000 mM NaCl) in a buffer containing 2 mM DTT, 2 mM EDTA, and 20 mM Pipes, pH 6.5. Fractions were analyzed by SDS-PAGE with Coomassie staining. Samples for SDS-PAGE were prepared in a loading buffer containing 5% 2-mercaptoethanol, 10% sucrose, 1.5 mM bromophenol blue, 62.5 mM Tris, pH 6.5, and 4% SDS. Fractions with the highest Tau content were pooled and further purified by size exclusion chromatography on a Superdex 200 (XK26/100) column, eluting with a buffer containing 2 mM DTT, 1 mM EDTA, 20 mM Tris (pH 7.4), and 100 mM NaCl. Fractions were again analyzed by SDS-PAGE, and those containing pure Tau were pooled. The protein was transferred into Spectra/Por tubing (6000–8000 Da cutoff) and dialyzed against a phosphate buffer (2 mM DTT, 10 mM sodium phosphate, pH 7.4) with a total of three buffer exchanges. Purified protein was aliquoted into 1 mL portions, flash frozen in liquid nitrogen, and stored at −80 °C until further use.

### Demographics of Brain Tissue Cases

Brain tissues from 12 post-mortem cases were used for the study (Table S1). Three cases from each condition (Control, AD, PiD, and PSP) were used. The tissues were provided by the Carrol A. Campbell, Jr. Neuropathology Lab at the Medical University of South Carolina (MUSC) and by the University of California Alzheimer’s Disease Research Center (UCI-ADRC) and the Institute for Memory Impairments and Neurological Disorders. Each case underwent expert neuropathological evaluations following post-mortem donation. This included assessment of AD and other pathology according to current staging paradigms (see *e*.*g*.,^71–73^ ). The average age for the control group was 59 years of age (YO), for the AD it was 81 YO, for the PiD it was 71 YO, and for the PSP group it was 69 YO. Although there was a significant difference between these groups in terms of age (F (3, 8) = 40.24; *p* < 0.0001) and in terms of *post-mortem* interval (PMI; F (3, 8) = 5.866; *p* = 0.0203), these differences had no effect on the outcome of the experiments. Due to the nature and progression of these different neurological disorders, the age at death was lower in the PiD and PSP groups than in the AD group, which was composed of late onset (LOAD), sporadic AD cases only. Detailed demographics for the tissue used in the experiments are listed in Table S1. There were no effects of age, gender, or PMI on any of the outcome measures.

### Brain Tissue Homogenization

Frozen human brain tissue (AD, PiD, PSP, and controls; see Table S1) was combined at a 1:10 (w/v) ratio with a buffer containing 10 mM HEPES, pH 7.4, 5 mM EDTA, 150 mM NaCl, and 0.1% Triton X-100, supplemented with 1× Halt Protease Inhibitor Cocktail (Thermo Scientific). The mixture was then homogenized on ice for 5 min in a 10 mL Potter-Elv tissue grinder, sonicated in a Fisher Scientific bath sonicator for 2 min at power setting 2, followed by centrifugation at 3000*g* for 10 min and collection of supernatants. Total protein concentrations of all supernatants were determined by the BCA assay. Samples were adjusted to 3 mg/mL, aliquoted, flash frozen, and stored at −80 °C until further use.

### Amplification of Pathological Tau Fibrils (Generation 1)

To amplify Tau fibrils, recombinant 3R or 4R Tau monomers (10 μM), were mixed with homogenized human brain tissue using biological triplicates (AD 1–3, PiD 1–3, PSP, 1–3, Control 1–3; diluted 1:100 to a final concentration of 30 μg/mL) in reaction buffer containing 5 μM Thioflavin T (ThT), 10 mM DTT, and 10 mM sodium phosphate, pH 7.4 (total volume = 400 μL). At this dilution, the mass-to-mass ratios of 3R and 4R Tau to total brain protein were 12:1 and 15:1, respectively. The samples were briefly vortexed (5–10 s), dispensed in triplicate (100 μL) into a 96-well optical-bottom polymer base plate (Thermo Scientific, catalogue no. 265301), closed with a sterile foil sealing film (Celltreat), and monitored in a BMG FLUOstar Omega plate reader, shaking for 1 min at 400 rpm every 10 min until completion. Fluorescence was recorded through the bottom of the plate with the excitation set at 440 nm and emission set at 480 nm.

To further dissect the specific conditions required for fibril amplification, additional control experiments were carried out in which a single reaction parameter was altered: (1) Effects of ThT: reaction buffer without ThT; (2) Ionic strength: reaction buffer containing 150 mM NaCl; (3) Oxidation status of Tau protein: incubation with oxidized 3R Tau dimers or compact 4R Tau monomers (see subsection “Preparation of Oxidized Tau”) without DTT; and (4) Homogenate-free aggregation: incubation of 3R or 4R Tau monomers in the absence of brain homogenates.

### Serial Amplification of Tau Fibrils (Generation 2 to 6)

Reaction mixtures containing Generation 1 fibrils, amplified from brain homogenates, were recovered from 96-well polymer base plates. The technical replicates of each biological replicate were pooled and transferred into 2 mL tubes. Samples were diluted 1:10 in phosphate buffer, pH 7.4 (400 μL) and tip-sonicated on ice for 30 s at a power setting of 2 in a Fisher Scientific Sonifier (equipped with a 2 mm tip) to generate small fibril seeds. The resulting suspension was then again diluted 1:10 into a fresh reaction buffer containing recombinant Tau monomers (10 μM). For this new reaction, fibril seeds from Generation 1 were thus diluted to 1:100. The mixtures were dispensed into 96-well polymer base plates, and fibril elongation was monitored in a FLUOstar plate reader using the same protocol described above. This amplification cycle resulted in Generation 2 fibrils. The fibrils were again harvested, diluted, and the tip-sonicated as described above. The resulting seeds were introduced into a new monomer reaction mixture under identical buffer and assay conditions to form the Generation 3 fibrils. This serial seeding process was repeated as needed, with each round of amplification producing progressively propagated fibril generations that were continuously monitored by ThT fluorescence. The effect of salt on serial amplification was tested by diluting fibril seeds from Generation 1 either 1:100 (1% seeds) or 1:10 (10% seeds) in 10 mM phosphate buffer (pH 7.4) containing 150 mM NaCl (final concentration) and incubating the samples for 77 h at 37 °C using the RT-QuIC protocol (shaking for 1 min at 400 rpm every 10 min) followed by sedimentation analysis.

### Testing for Cross-Seeding Barriers

Whenever cross-seeding barriers were probed, the Tau monomers employed in the amplification reaction were different from the ones that generated the fibril seeds in the preceding reaction, i.e., 3R Tau monomers were used on 4R Tau seeds and vice versa.

### Sedimentation Assay

After each round of amplification, the samples were analyzed for their degree of aggregation. For this purpose, samples (nonsonicated) were centrifuged for 30 min at 400,000*g* (4 °C) and separated into pellets and supernatants. The two fractions were taken up in SDS sample buffer and adjusted in volume to allow for a comparison of equivalent amounts by SDS-PAGE and Coomassie staining. The band intensities were quantified by ImageJ software (National Institutes of Health). For this purpose, the band intensities of each pellet and supernatant were divided by the total band intensities (pellet plus supernatant), multiplied by 100, and then plotted using GraphPadPrism 7 software.

### Preparation of Oxidized Tau

3R and 4R Tau monomers were oxidized for 24 h at 22 °C with 1 mM hydrogen peroxide in a buffer containing 40 mM HEPES, pH 8.2, and 100 mM NaCl. This generated dimers of 3R Tau with intermolecular disulfide bonds between the single cysteines at position 291; and compact monomers of 4R Tau with intramolecular disulfide bonds between the two cysteines at positions 291 and 322.^68,74^ The proteins were purified by size exclusion chromatography (Superdex 200 10/300 GL column) and dialyzed against 10 mM sodium phosphate buffer, pH 7.4. The Oxidation status of Tau was confirmed by nonreducing SDS-PAGE for 3R Tau (2-mercaptoethanol was absent in the loading buffer) and native gel electrophoresis for 4R Tau.^75^

### Transmission Electron Microscopy

Samples of the reaction mixtures for TEM evaluation were diluted to 5 μM. A 10 μL drop of the mixture was placed onto Formvar/carbon-coated 200 mesh copper grids (Electron Microscopy Sciences) for 60 s. The side of the grid was then lightly tapped on filter paper to remove the extra liquid. A 10 μL drop of 2% uranyl acetate was placed onto the grid for 60 s, and the grid was dried again. Images were collected on an FEI Tecnai T12 BioTwin electron microscope at 100 keV equipped with a Gatan CCD camera.

### Tau Aggregation in Cell Culture

Monoclonal 4R Tau-P301S-EYFP HEK293 cells^49^ were plated at 10,000–20,000 cells per well in a 96-well glass-bottom plate (Greiner, catalogue# 655891) containing 10% FBS in DMEM and 700 μg/mL G418 (total volume of 200 μL). Cells grew until they reached a confluency of 80–100%. Seeds from the sonicated reaction mixtures were combined with Lipofectamine 2000 and Opti-MEM, incubated for 5 min, and then added to the cells at a final seed concentration of 1 μM. Buffer (10 mM sodium phosphate, pH 7.4) mixed with Lipofectamine 2000 and Opti-MEM was used as a control. Cells were incubated for 24 h at 37 °C and then imaged using an Olympus FluoView FV3000 Confocal Microscope equipped with a 488 nm laser.

### Dynamic Light Scattering

3R or 4R Tau monomers were prepared at a final concentration of 10 μM in 10 mM sodium phosphate buffer (pH 7.4) either with or without 150 mM NaCl (final concentration). Samples were incubated for 2 h at 22 °C, followed by a 15 min spin at 15,000*g* through an Amicon Ultra-0.5 centrifugal filter device with 100,000 Molecular Weight Cutoff (Millipore Sigma, catalogue no. UFC510096) and dispensed (100 μL) into a 96-well optical-bottom polymer base plate (Thermo Scientific, catalogue# 265301). Dynamic light scattering data were collected using a Wyatt Technology DynaPro II Plate Reader at a wavelength of 830 nm; experimental parameters were set to 20 acquisitions for 5 s each at 25 °C. Data acquired from the plate reader were then analyzed using Dynamics7 software by Wyatt Technology.

## Supplementary Material



## Notes

Figures with sedimentation data (SDS-PAGE gels), aggregation control experiments, dynamic light scattering of monomers, electron micrographs of amplified fibrils and seeds, replicate HEK293 cell images, as well as tables containing information regarding human brain tissue and HEK293 cell quantifications (PDF)
