# Supplementary material for: Cofactor-Free Serial Amplification of Tau Filaments from Alzheimer’s Disease and Other Tauopathies Depends on the Conformational State of Tau Monomers
Source: JACS Au. 2026 Mar 11;6(3):1789–800. doi: 10.1021/jacsau.5c01693 (PMC13014239; doi:10.1021/jacsau.5c01693)
Supplement: Supplementary file 1 [file au5c01693_si_001.pdf]

Cofactor-Free Serial Amplification of Tau Filaments from Alzheimer's Disease and Other Tauopathies Depends on the Conformational State of Tau Monomers

Zachariah Y. Gabani[1‡], Jasdeep Singh[1‡], Eric D. Hamlett[2], Ann-Charlotte Granholm[3], and Martin Margittai[1]*

[1]Department of Chemistry and Biochemistry, University of Denver, Denver, CO 80208, USA

[2] Department of Pathology and Laboratory Medicine, Medical University of South Carolina, Charleston, SC 29425, USA

[3] Department of Neurosurgery, University of Colorado Anschutz, Aurora, CO 80045, USA

[‡]These authors contributed equally

*To whom correspondence should be addressed: Martin Margittai, Department of Chemistry and Biochemistry, University of Denver, 2190 East Iliff Ave, Denver, CO 80208. Tel: (303)-871-4135; Fax: (303)-871-2254. E-mail: martin.margittai@du.edu

Contents

Figure S1. Full-length 3R and 4R Tau monomers aggregate in the presence of AD brain homogenates.
Figure S2. Full-length 3R and 4R Tau monomers do not aggregate in the absence of brain homogenate.
Figure S3. AD brain homogenates convert full-length 3R and 4R Tau monomers into aggregates in the absence of ThT.
Figure S4. AD brain homogenates fail to convert full-length 3R and 4R Tau monomers into aggregates at 150 mM NaCl.
Figure S5. Tau fibrils amplified from AD brain homogenates do not dissociate in the presence of 150 mM NaCl.
Figure S6. Tau monomers in 150 mM NaCl have a smaller hydrodynamic radius than in its absence.
Figure S7. AD brain homogenates fail to convert oxidized 3R and 4R Tau into aggregates.
Figure S8. AD Generation 1 fibril seeds used for serial amplification have similar lengths.
Figure S9. Serial amplification of AD fibrils with recombinant 3R Tau does not require cofactors.
Figure S10. Serial amplification of AD fibrils with recombinant 4R Tau does not require cofactors.
Figure S11. Generation 1 fibrils from AD do not amplify in the presence of 150 mM NaCl.
Figure S12. Tau Aggregates generated by serial amplification are fibrillar.
Figure S13. Serially amplified Tau fibrils retain cross-seeding abilities.
Figure S14. Tau fibrils amplified from AD brain homogenates induce intracellular Tau aggregation.
Figure S15. Homogenates from PiD and PSP convert 3R and 4R Tau monomers into aggregates, respectively.
Figure S16. PiD and PSP brain homogenates fail to convert Tau monomers when 150 mM NaCl is added.
Figure S17. Generation 1 seeds from PiD and PSP have similar lengths.
Figure S18. Generation 1 Tau fibrils from PiD and PSP can be serially amplified while retaining their characteristic cross-seeding properties.
Table S1. Brain tissue used in this study.
Table S2. Cell and puncta count after seed treatment of HEK293 cells.

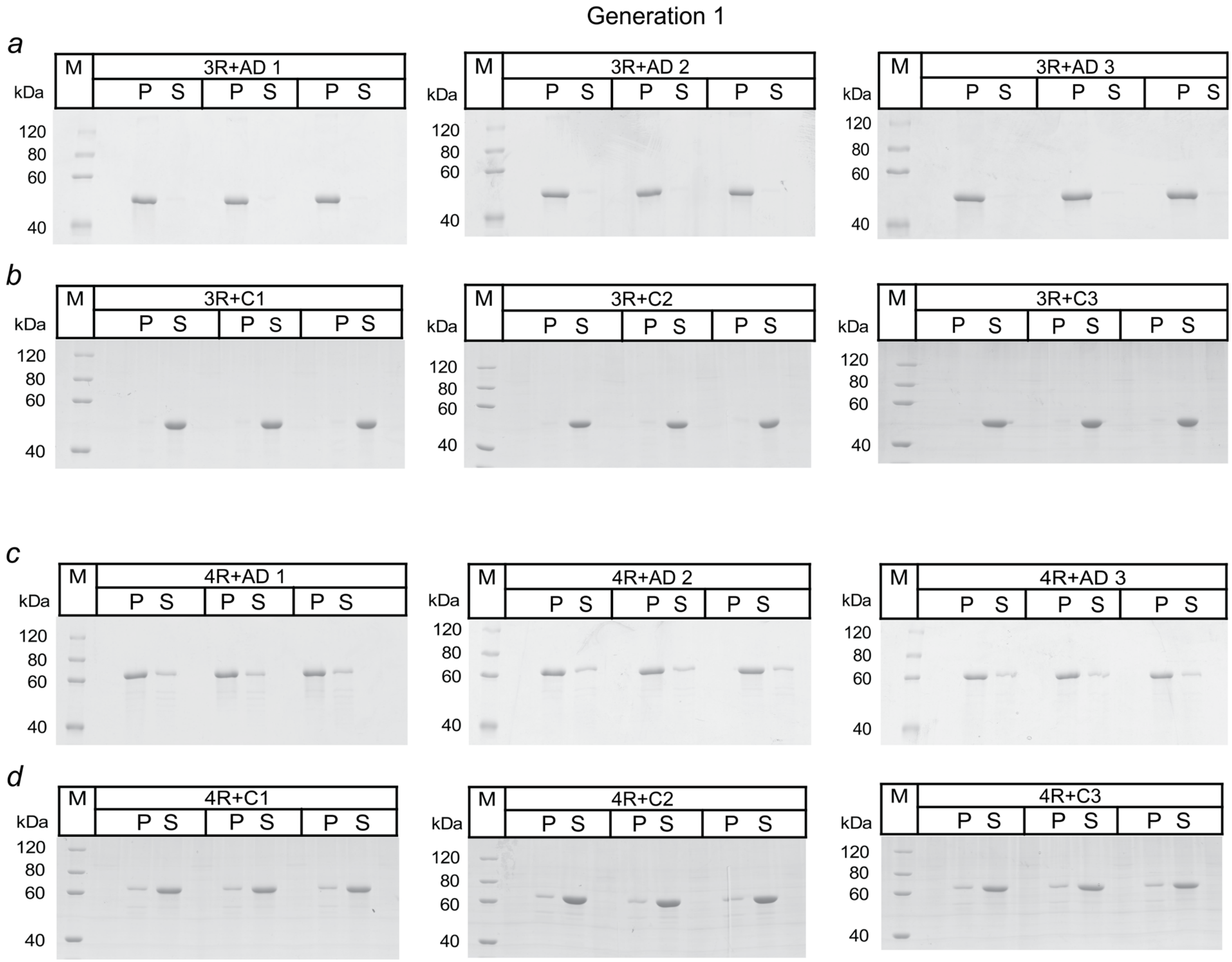

Figure S1. Full-length 3R and 4R Tau monomers aggregate in the presence of AD brain homogenates. After Tau monomers were mixed with AD (AD 1-3) or control (C1-3) brain homogenates and subjected to the RT-QuIC protocol (Fig. 1 a, c), the reactions were sedimented and analyzed by SDS-PAGE and Coomassie staining. **(a)** 3R Tau + AD 1-3. **(b)** 3R Tau + C1-3. **(c)** 4R Tau + AD 1-3. **(d)** 4R Tau + C1-3. Each experiment was carried out in triplicate. P, pellet; S, supernatant. M, protein marker. 3R Tau runs at an apparent molecular weight of 52 kDa; 4R Tau runs at 64 kDa. The data were used to quantify the distribution of Tau protein into pellets versus supernatants (Fig. 1 b, d).

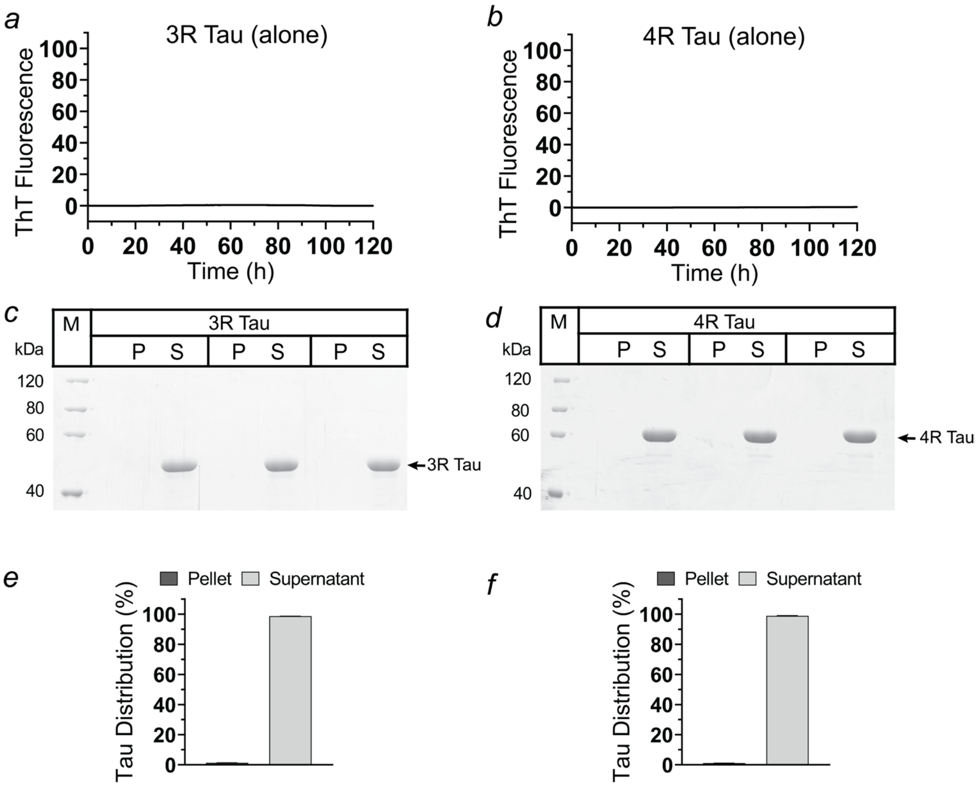

Figure S2. Full-length 3R and 4R Tau monomers do not aggregate in the absence of brain homogenate. Recombinant Tau monomers (10 µM) were incubated for 120 h at 37°C using RT-QuIC. ThT traces for 3R Tau **(a)** and 4R Tau **(b)**. The samples were sedimented and analyzed by SDS-PAGE and Coomassie staining. Gels for 3R Tau **(c)** and 4R Tau **(d)**. M, protein marker. The band intensities of pellets (P) and supernatants (S) were quantified and graphed. Graphs for 3R Tau **(e)** and 4R Tau **(f)**. All experiments were carried out in triplicate. Error bars represent means ± SD.

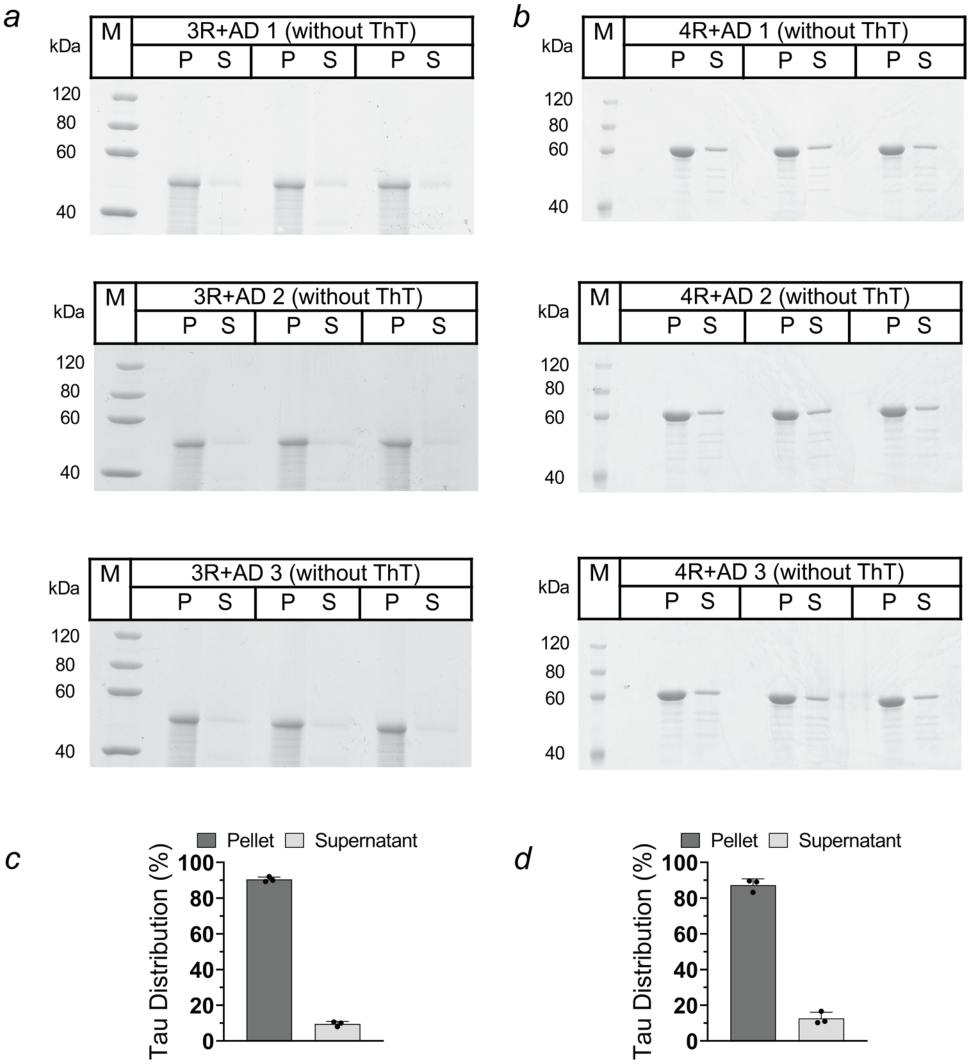

Figure S3. AD brain homogenates convert full-length 3R and 4R Tau monomers into aggregates in the absence of ThT. Tau monomers were mixed with AD brain homogenates as before, but without the inclusion of ThT. The samples were then incubated for 90 h at 37 ºC using RT-QuIC, sedimented, and analyzed by SDS-PAGE and Coomassie staining. **(a)** 3R Tau + AD 1-3. **(b)** 4R Tau + AD 1-3. Each of the biological replicates was loaded in triplicate (n=3). M, protein marker. The band intensities of pellets (P) and supernatants (S) were quantified by densitometry and plotted. Bar graphs for 3R Tau **(c)** and 4R Tau **(d)**. Error bars represent means ± SD.

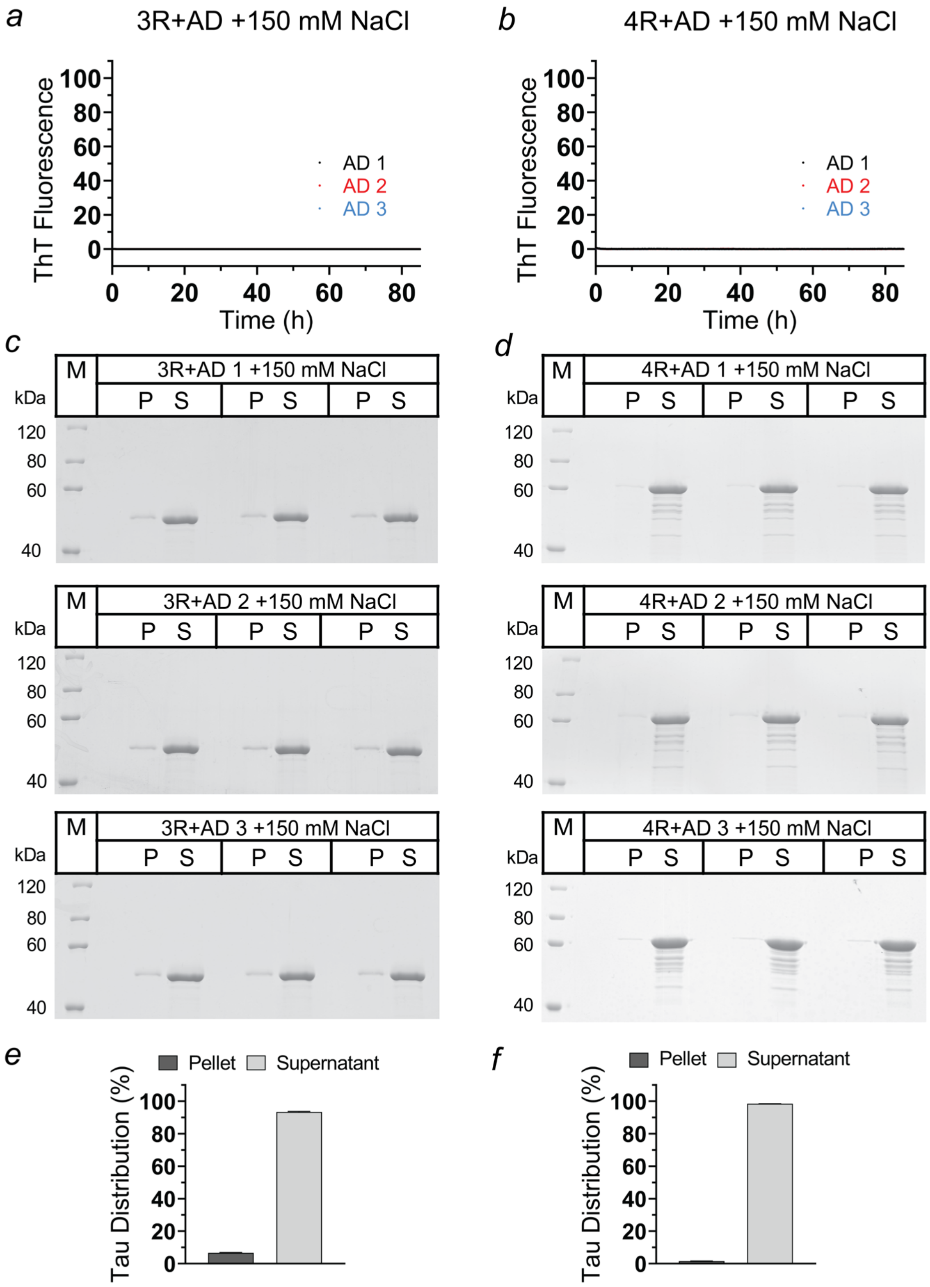

Figure S4. AD brain homogenates fail to convert full-length 3R and 4R Tau monomers into aggregates at 150 mM NaCl. Recombinant Tau monomers (10 µM) were mixed with AD (AD 1-3) brain homogenates (30 µg/mL) in 10 mM sodium phosphate buffer (pH 7.4) containing 150 mM NaCl and incubated at 37 ºC using RT-QuIC. ThT traces of reactions with 3R Tau **(a)** and 4R Tau monomers **(b)**. The samples were sedimented and analyzed by SDS-PAGE and Coomassie staining. Gels for 3R Tau **(c)** and 4R Tau **(d)**. M, protein marker. Band intensities of pellets (P) and supernatants (S) were quantified and graphed for reactions involving 3R Tau **(e)** and 4R Tau monomers **(f)**. All experiments were carried out in triplicate. Error bars represent means ± SD.

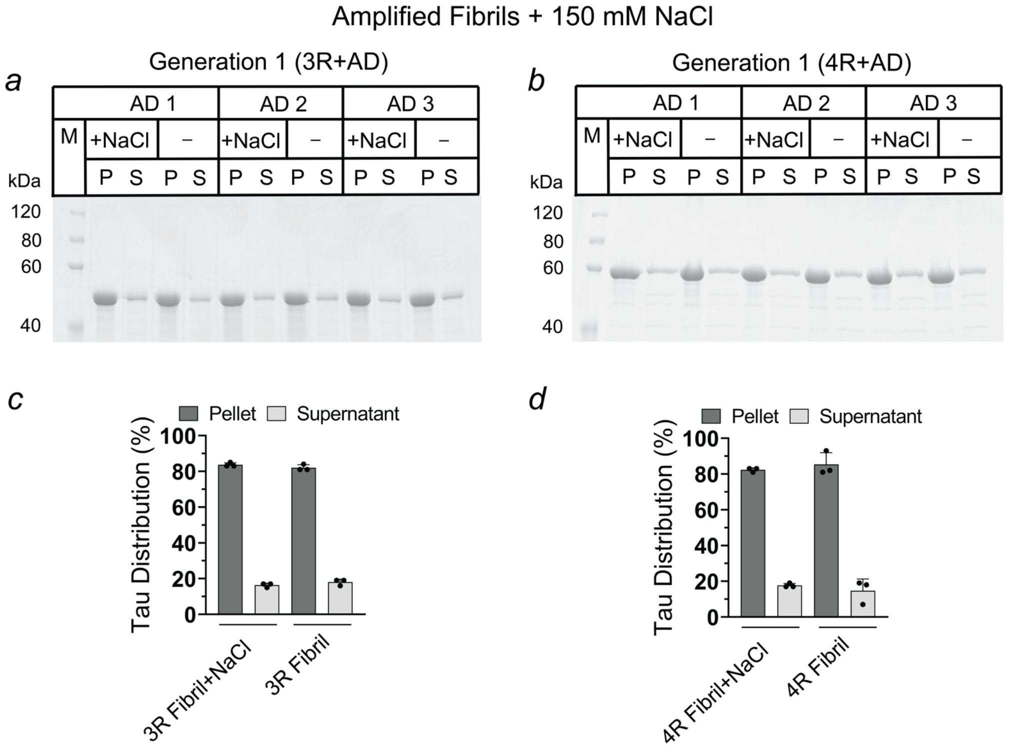

Figure S5. Tau fibrils amplified from AD brain homogenates do not dissociate in the presence of 150 mM NaCl. Generation 1 fibrils (10 µM monomer equivalents) formed in 10 mM phosphate buffer (pH 7.4) were combined with a final concentration of 150 mM NaCl or an equivalent volume of phosphate buffer (3% v/v) as control and quiescently incubated for 24 h at 22 °C. The samples were then sedimented and analyzed by SDS-PAGE and Coomassie staining. **(a)** 3R Tau +AD 1-3. **(b)** 4R Tau + AD 1-3. AD 1-3 are biological replicates. M, protein marker. NaCl, buffer adjusted to 150 mM NaCl; minus (-), no NaCl added. The band intensities of pellets (P) and supernatants (S) were quantified by densitometry and plotted. Bar graphs for 3R Tau **(c)** and 4R Tau **(d)** reactions. Error bars represent means ± SD.

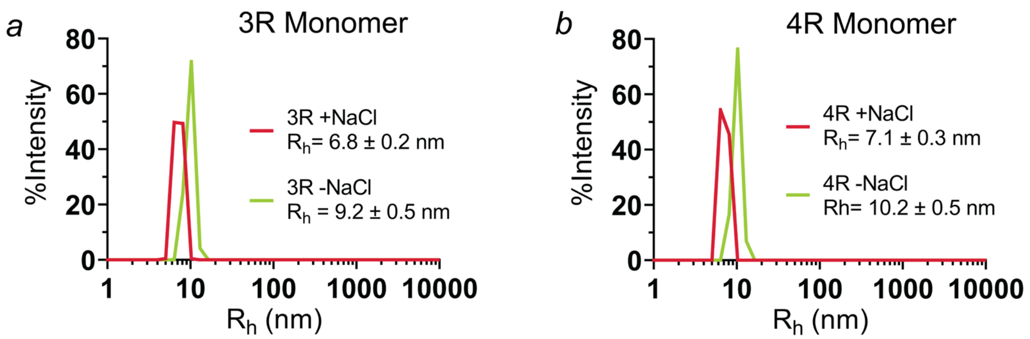

Figure S6. Tau monomers in 150 mM NaCl have a smaller hydrodynamic radius than in its absence. Tau monomers were diluted to a final concentration of 10 µM, supplemented with 10 mM sodium phosphate buffer (pH 7.4) in the presence or absence of 150 mM NaCl, filtered and analyzed by dynamic light scattering. Size distributions of 3R Tau **(a)** and 4R Tau monomers **(b)** in the presence (red) or absence (green) of 150 mM NaCl. R_h, hydrodynamic radius. Curves represent the averages of 20 acquisitions (5 s each).

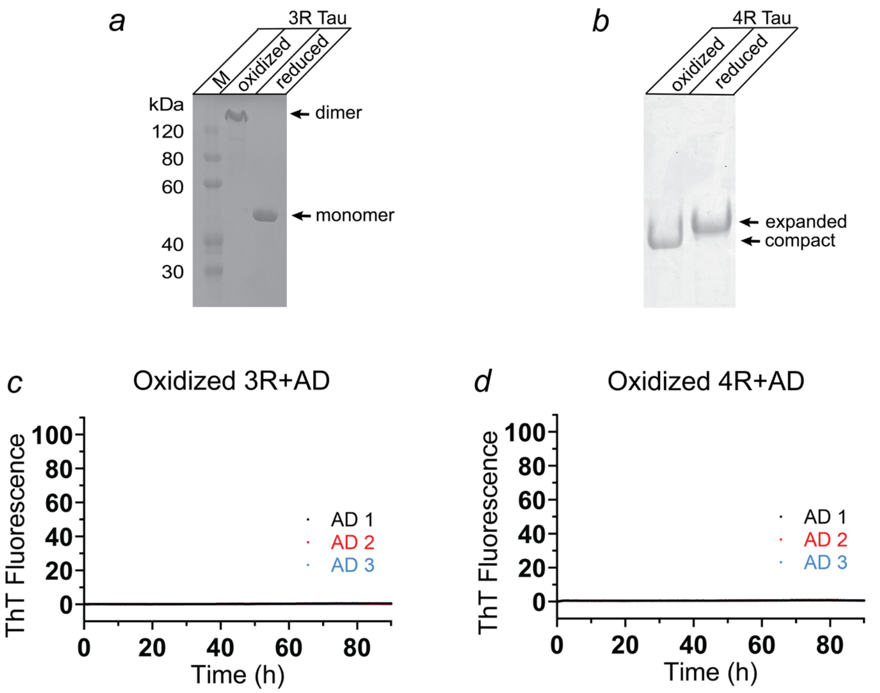

Figure S7. AD brain homogenates fail to convert oxidized 3R and 4R Tau into aggregates. Tau was oxidized with 1 mM hydrogen peroxide to generate intermolecular disulfide linkages between the single cysteines (position 291) of two 3R Tau monomers and intramolecular disulfide linkages between the two cysteines (positions 291 and 322) of one 4R Tau monomer. The proteins were purified by size exclusion chromatography. Oxidized 3R Tau was analyzed by non-reducing SDS-PAGE to identify dimers **(a)**. Oxidized 4R Tau was analyzed by Native-PAGE to detect compact monomers **(b)**. The reduced forms of the proteins (with free thiol groups) were run as controls. Oxidized Tau (10 µM) was then mixed with AD (AD 1-3) brain homogenates (30 µg/mL) in 10 mM sodium phosphate buffer (pH 7.4), free of reducing agent and incubated at 37 ºC using the RT-QuIC protocol. ThT traces of reactions with oxidized 3R Tau **(c)** and oxidized 4R Tau **(d)**. All experiments were carried out in triplicate. Error bars (not visible here) represent means ± SD.

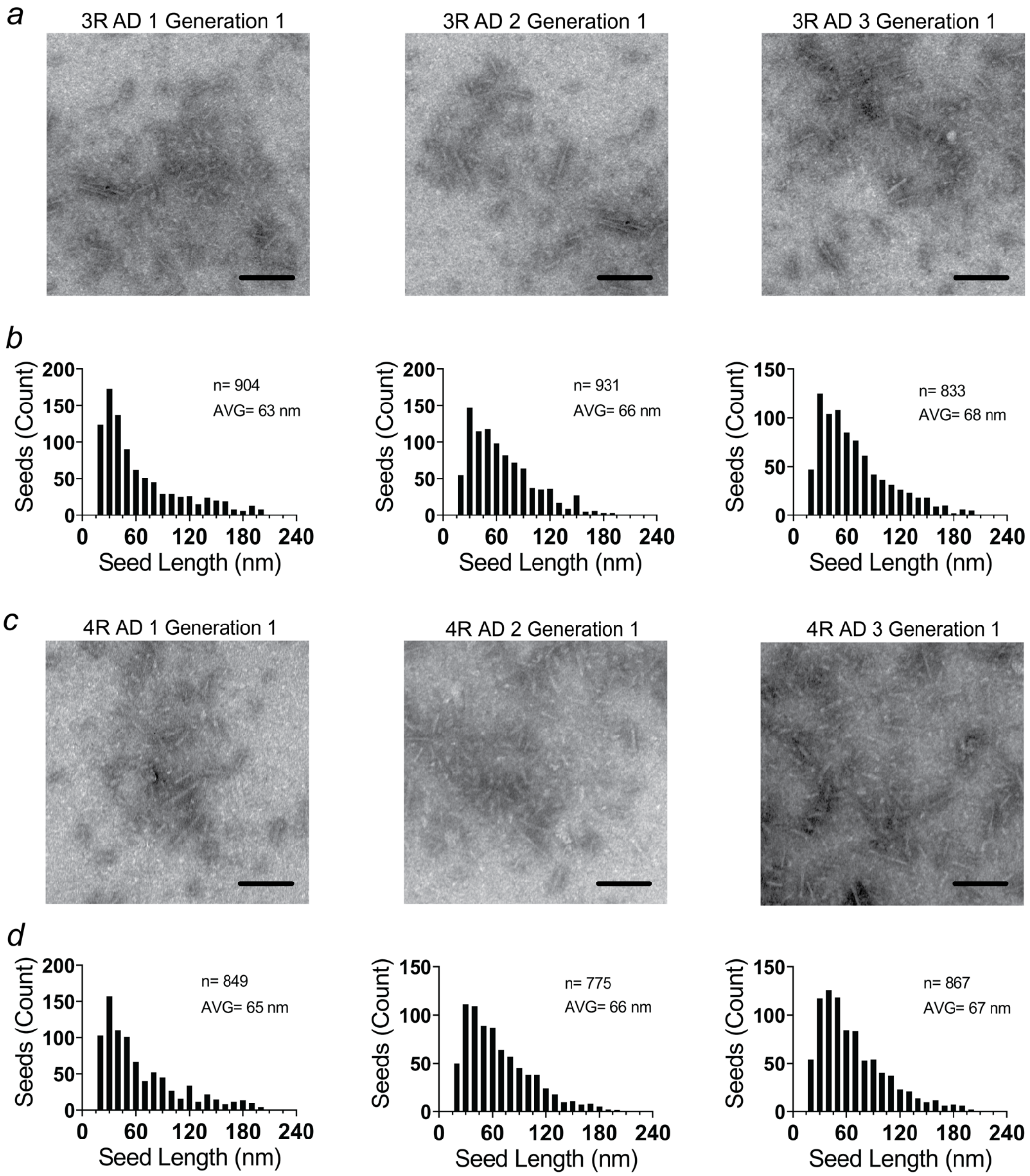

Figure S8. AD Generation 1 fibril seeds used for serial amplification have similar lengths. AD 1-3 fibrils generated by a first round of amplification (Generation 1) with either 3R or 4R Tau monomers (Fig. 1) were tip-sonicated for 30 s on ice and imaged by negative stain transmission electron microscopy. Representative images of seeds composed of 3R Tau **(a)** and quantification **(b)**. Representative images of seeds composed of 4R Tau **(c)** and quantification **(d)**. Left, center, and right panels represent data for seeds that descended from AD 1, AD 2, and AD 3 homogenates, respectively. Scale bars, 100 nm.

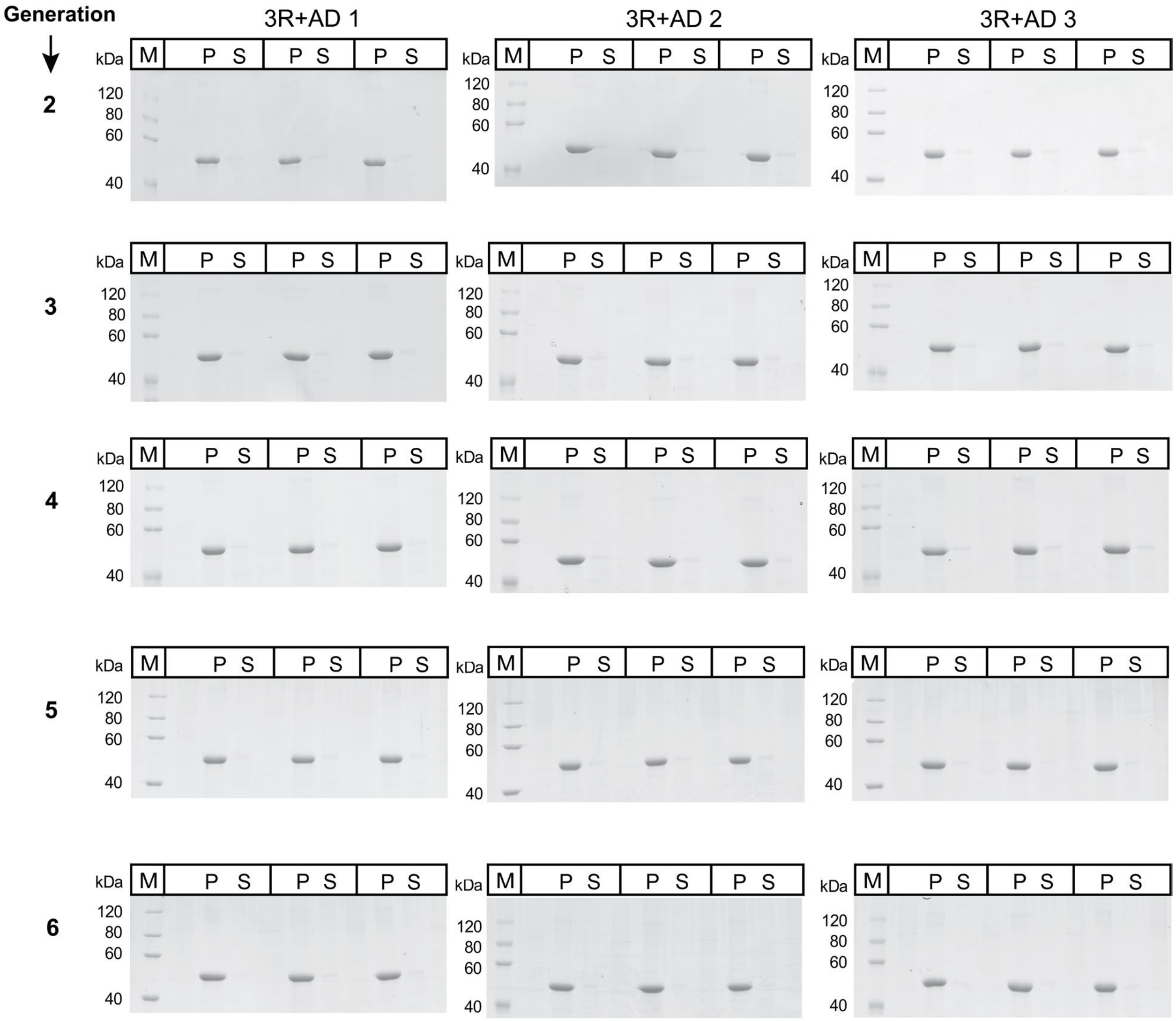

Figure S9. Serial amplification of AD fibrils with recombinant 3R Tau does not require cofactors. AD fibrils (Generation 2-6) that were amplified with 3R Tau monomers using the RT-QuIC protocol (Fig. 2a), were sedimented and analyzed by SDS-PAGE and Coomassie staining. Gels within a row represent biological triplicates, with seeds descended from the original AD brain homogenates (AD 1-3). Gels within a column represent the different rounds of amplification (Generation 2-6). M, protein marker. P, pellet; S, supernatant. 3R Tau runs at an apparent molecular weight of 52 kDa. The band intensities were used to quantify the distribution of Tau protein into pellets versus supernatants (Fig. 2b).

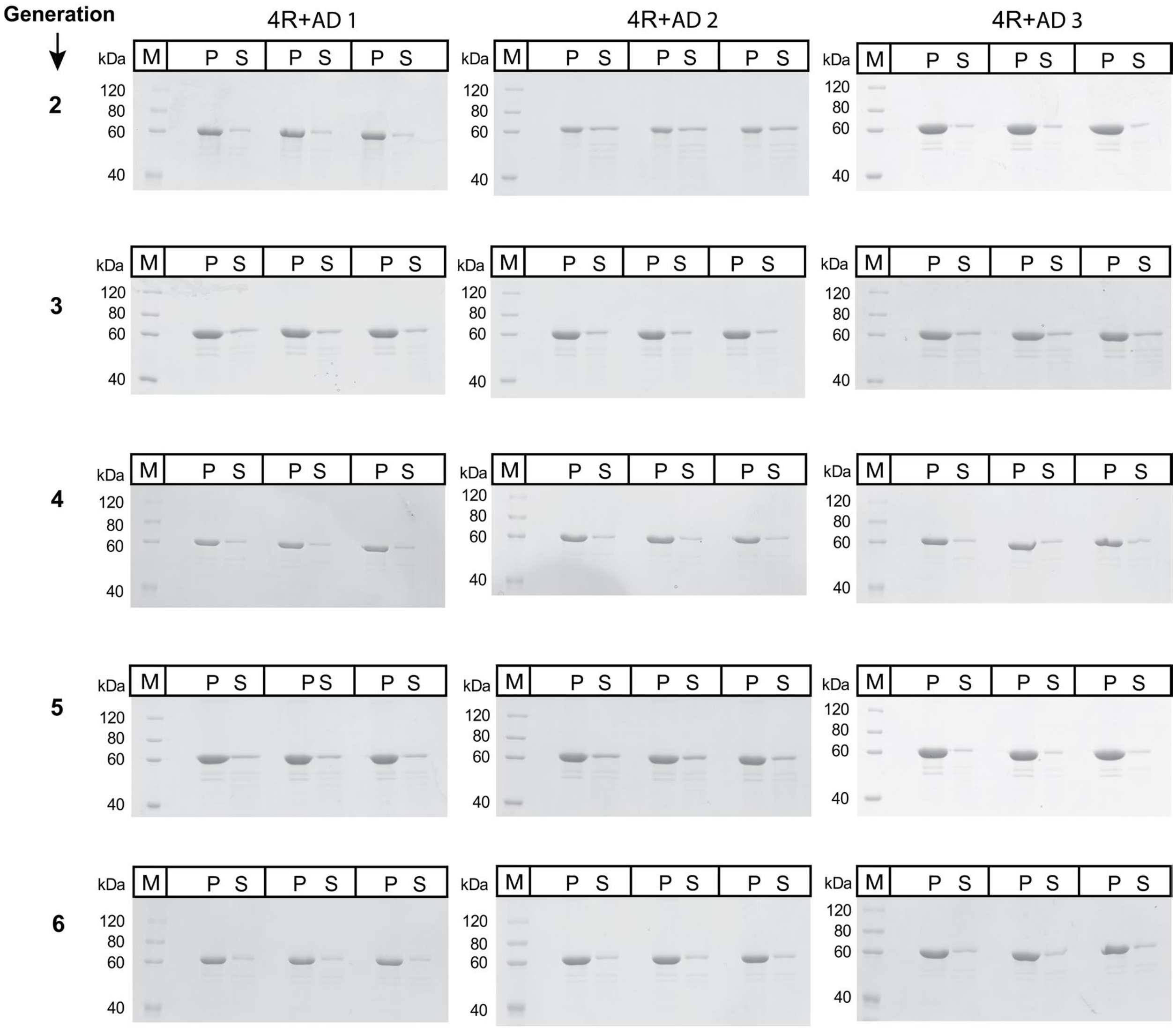

Figure S10. Serial amplification of AD fibrils with recombinant 4R Tau does not require cofactors. AD fibrils (Generation 2-6) that were amplified with 4R Tau monomers using the RT-QuIC protocol (Fig. 2c), were sedimented and analyzed by SDS-PAGE and Coomassie staining. Gels within a row represent biological triplicates, with seeds descended from the original AD brain homogenates (AD 1-3). Gels within a column represent the different rounds of amplification (Generation 2-6). M, protein marker. P, pellet; S, supernatant. 4R Tau runs at an apparent molecular weight of 64 kDa. The band intensities were used to quantify the distribution of Tau protein into pellets versus supernatants (Fig. 2d).

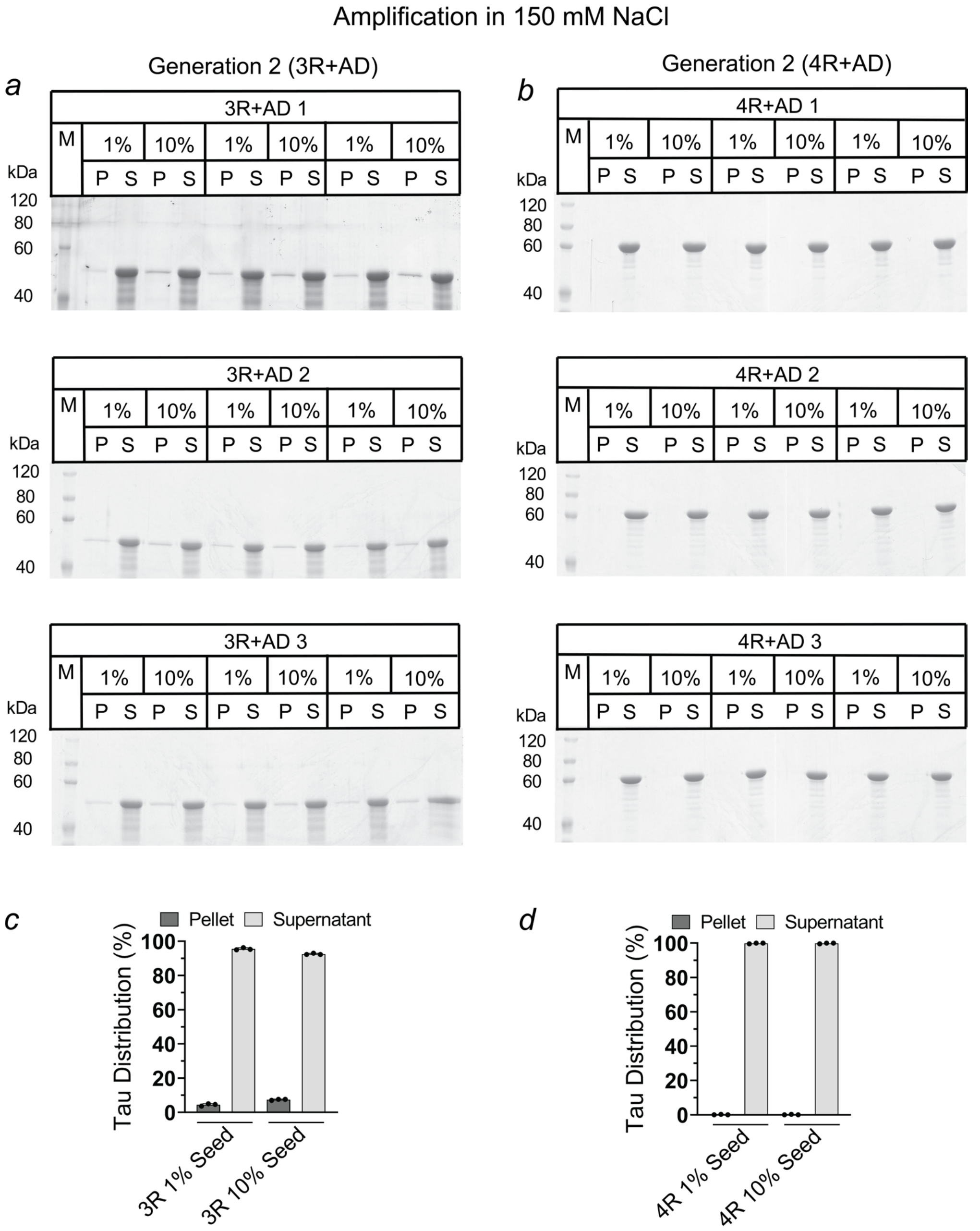

Figure S11. Generation 1 fibrils from AD do not amplify in the presence of 150 mM NaCl. Tau monomers (10 µM) were mixed with either 1% or 10% AD seeds (Generation 1) in 10 mM phosphate buffer and 150 mM NaCl. The samples were incubated for 77 h at 37 °C using RT-QuIC, sedimented, and analyzed by SDS-PAGE and Coomassie staining. **(a)** 3R Tau + AD 1-3. **(b)** 4R Tau + AD 1-3. Each of the biological replicates was loaded in triplicate (n=3). M, protein marker. The band intensities of pellets (P) and supernatants (S) were quantified by densitometry and plotted. Bar graphs for 3R Tau **(c)** and 4R Tau **(d)**. Error bars represent means ± SD.

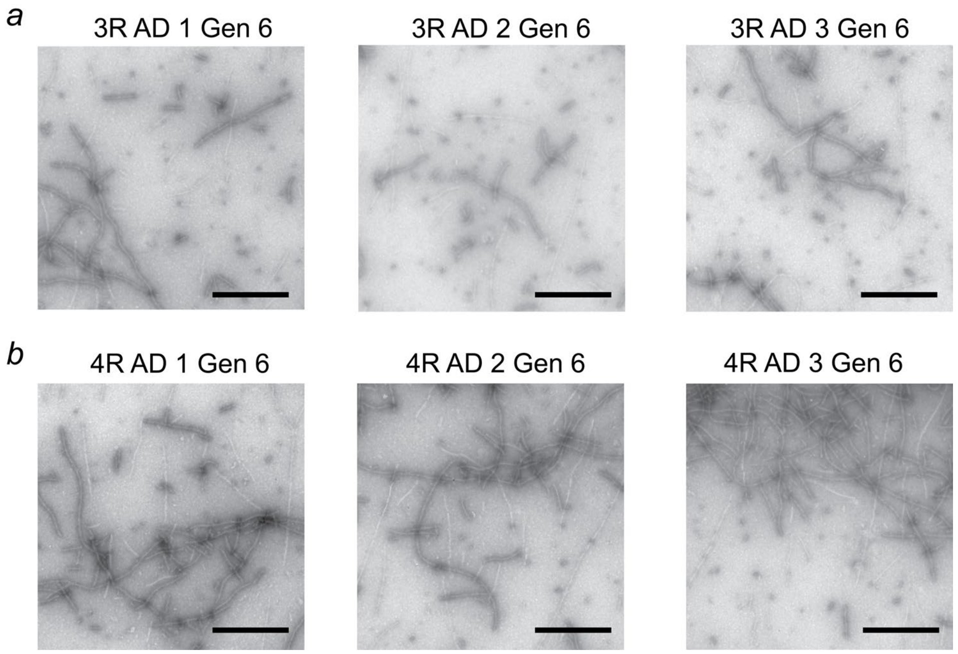

Figure S12. Tau aggregates generated by serial amplification are fibrillar. After the last round of amplification (Generation 6 in Figure 2), Tau aggregates were imaged by negative stain transmission electron microscopy. 3R Tau fibrils **(a)** and 4R Tau fibrils **(b)** descended from AD 1-3 brain homogenates. Scale bars, 500 nm.

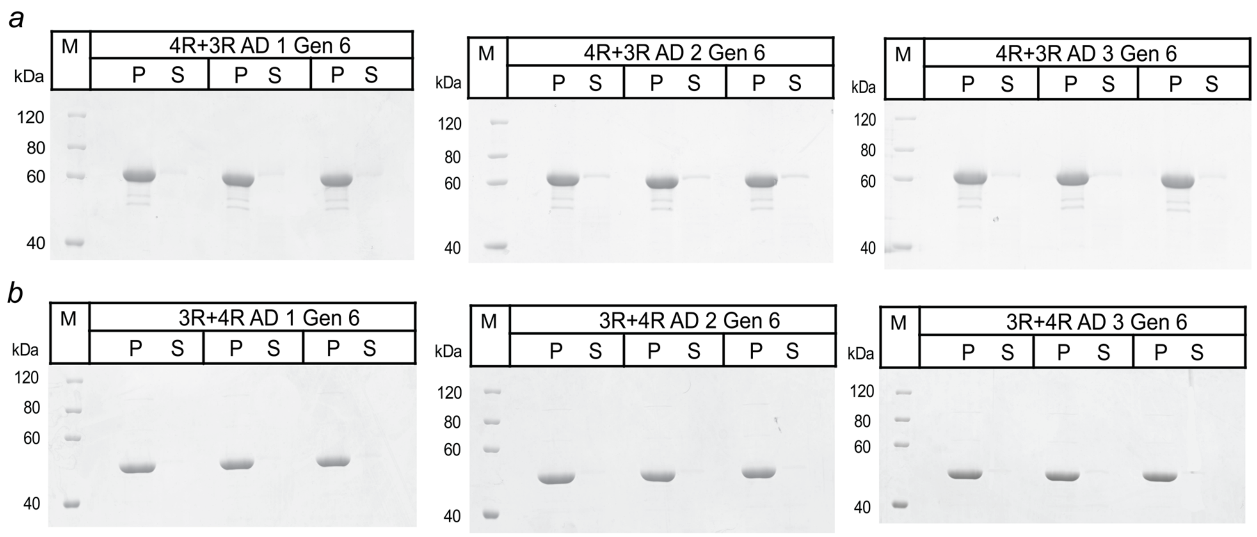

Figure S13. Serially amplified Tau fibrils retain cross-seeding abilities. Cross-seeded fibrils that were produced from Generation 6 fibrils using the RT-QuIC protocol (Fig. 3 a, c) were sedimented and analyzed by SDS-PAGE and Coomassie staining. Gels for reactions in which 4R Tau monomers were cross-seeded with 3R Tau fibrils **(a)** and 3R Tau monomers were cross-seeded with 4R Tau fibrils **(b)**. M, protein marker. P, pellet; S, supernatant. 3R Tau runs at an apparent molecular weight of 52 kDa; 4R Tau runs at 64 kDa. AD 1 (left), AD 2 (center), and AD 3 (right) signify the original AD brain homogenates the fibrils descended from. The band intensities were used to quantify the distribution of Tau protein into pellets versus supernatants (Fig. 3 b, d).

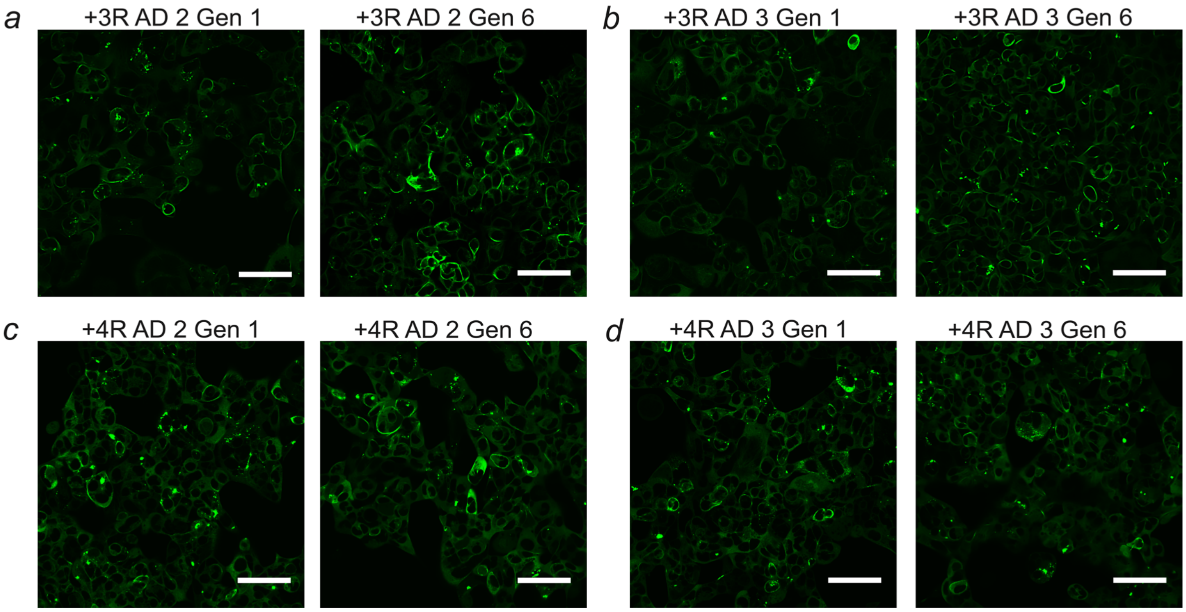

Figure S14. Tau fibrils amplified from AD brain homogenates induce intracellular Tau aggregation. Monoclonal HEK293 cells that expressed htau40P301S (a variant of 2N4R Tau) tagged with EYFP at the C-terminus were transfected with Tau seeds (Generations 1 and 6) and incubated for 24 h at 37 ºC. Representative images of cells transfected with AD 2-3 seeds amplified with 3R Tau monomers **(a-b)** or 4R Tau monomers **(c-d)**. Generation 1 (left panels). Generation 6 (right panels). Scale bars, 40 µm.

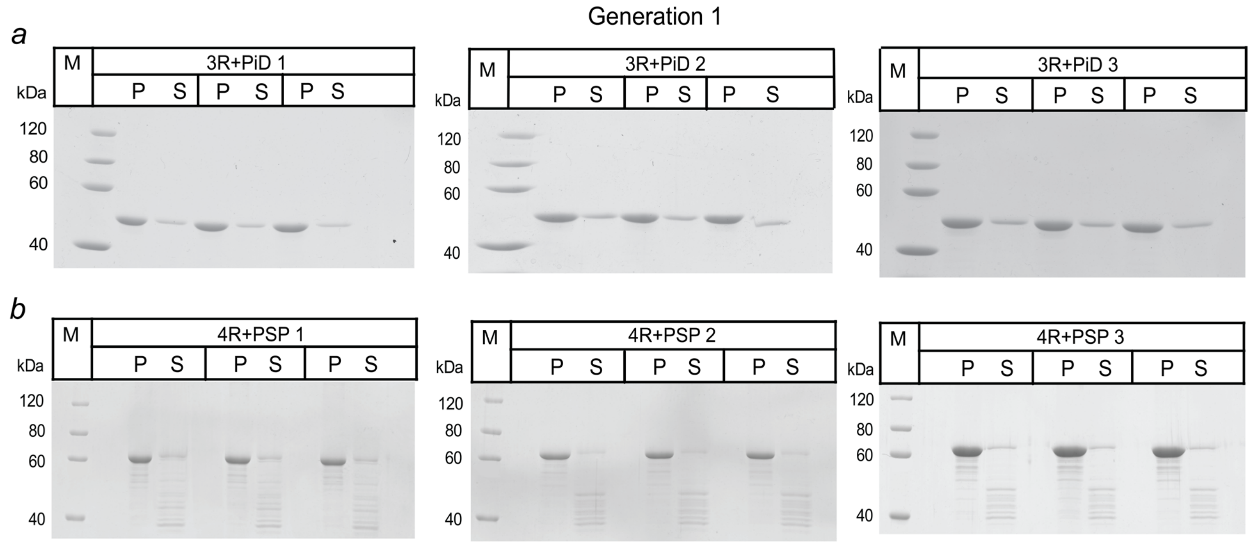

Figure S15. Homogenates from PiD and PSP convert 3R and 4R Tau monomers into aggregates, respectively. PiD fibrils and PSP fibrils (Generation 1) that were amplified with 3R Tau and 4R Tau monomers, respectively, using the RT-QuIC protocol (Fig. 5a, c), were sedimented and analyzed by SDS-PAGE and Coomassie staining. Gels for reactions seeded with PiD 1-3 homogenates **(a)** and PSP 1-3 homogenates **(b)**. M, protein marker. P, pellet; S, supernatant. The band intensities were used to quantify the distribution of Tau protein into pellets versus supernatants (Fig. 5b, d).

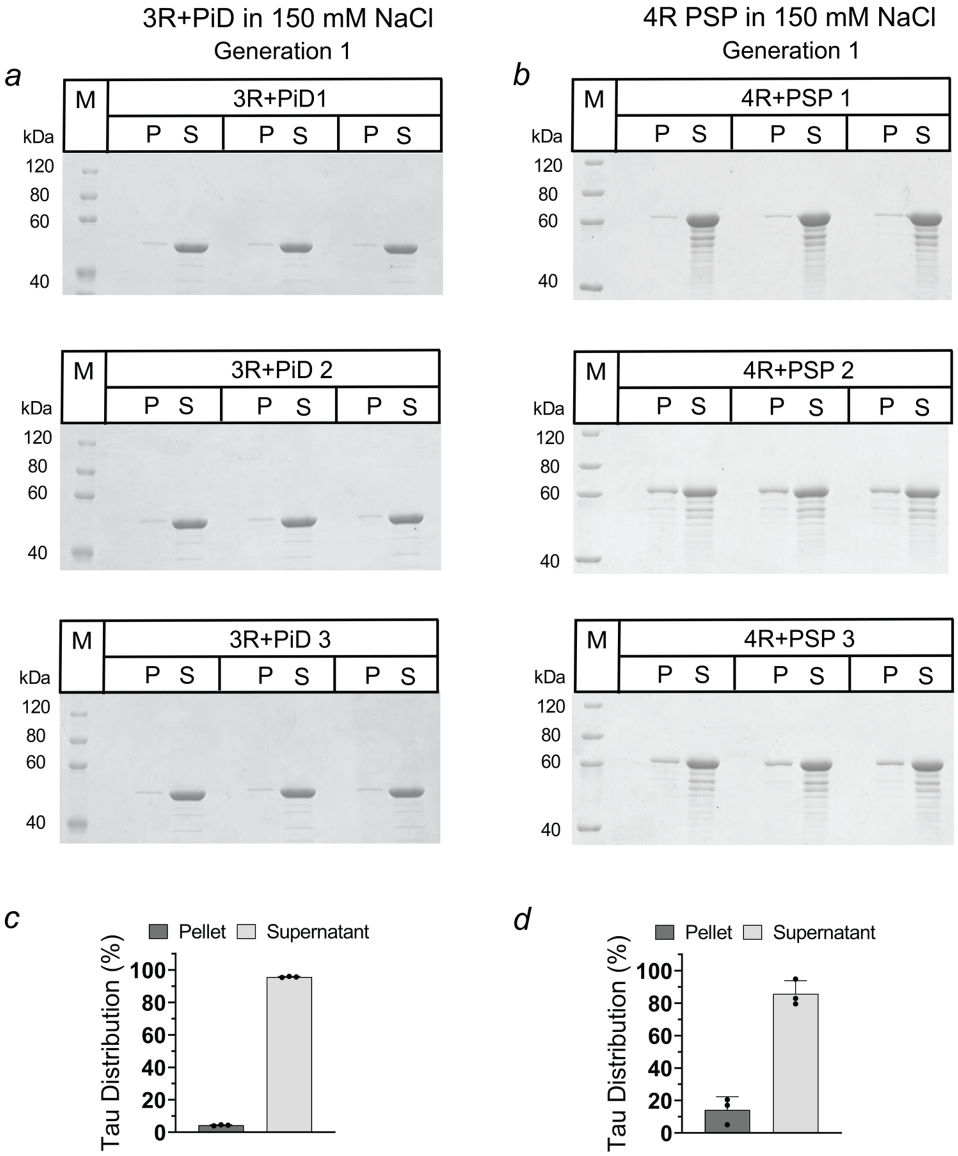

Figure S16. PiD and PSP brain homogenates fail to convert Tau monomers when 150 mM NaCl is added. Recombinant Tau monomers (10 μM) were mixed with PiD (PiD 1-3) or PSP (PSP 1-3) brain homogenates (30 μg/mL) in 10 mM sodium phosphate buffer (pH 7.4) containing 150 mM NaCl and incubated for 90 h at 37 ºC using the RT-QuIC protocol. The samples were sedimented and analyzed by SDS-PAGE and Coomassie staining. **(a)** 3R Tau + PiD 1-3. **(b)** 4R Tau + PSP 1-3. Each of the biological replicates was loaded in triplicate (n=3). M, protein marker. The band intensities of pellets (P) and supernatants (S) were quantified by densitometry and plotted. Bar graphs for 3R Tau **(c)** and 4R Tau **(d)**. Error bars represent means ± SD.

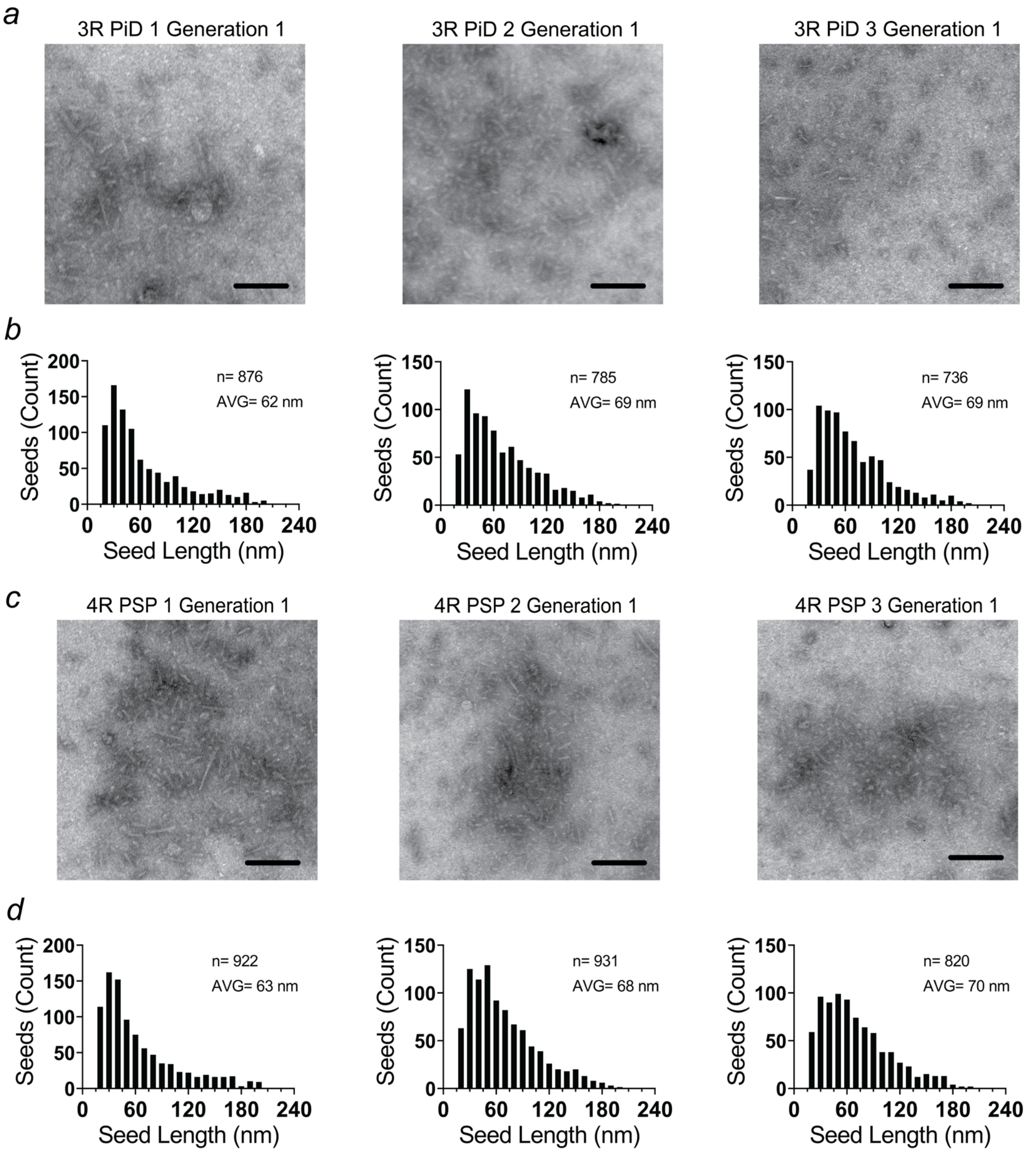

Figure S17. Generation 1 seeds from PiD and PSP have similar lengths. PiD 1-3 and PSP 1-3 fibrils generated by amplification (Generation 1) from brain homogenates mixed with 3R and 4R Tau monomers, respectively (Fig. 5 e-f), were tip-sonicated for 30 s on ice and imaged by negative stain transmission electron microscopy. Representative images of seeds from 3R Tau + PiD 1-3 **(a)** and quantification **(b)**. Representative images of seeds from 4R Tau + PSP 1-3 **(c)** and quantification **(d)**. Scale bars, 100 nm.

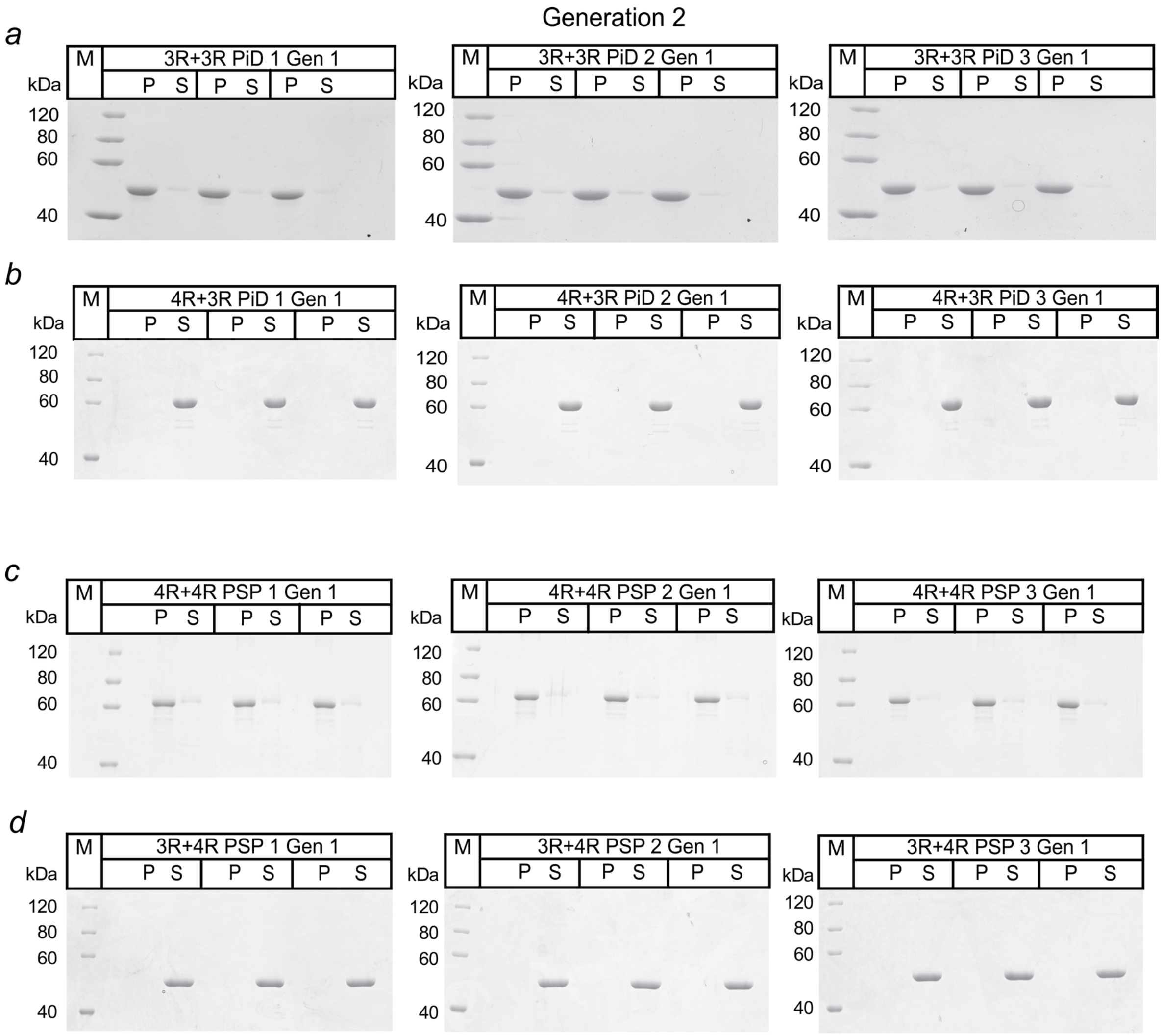

Figure S18. Generation 1 Tau fibrils from PiD and PSP can be serially amplified while retaining their characteristic cross-seeding properties. PiD 1-3 and PSP 1-3 fibrils that were serially amplified with 3R and 4R Tau monomers using the RT-QuIC protocol (Fig. 6a, c) were sedimented and analyzed by SDS-PAGE and Coomassie staining. Gels for reactions in which 3R Tau monomers were seeded with PiD fibrils **(a)** and 4R Tau monomers were cross-seeded with PiD fibrils **(b)**. Gels for reactions in which 4R Tau monomers were seeded with PSP fibrils **(c)** and 3R Tau monomers were cross-seeded with PSP fibrils **(d)**. M, protein marker. P, pellet; S, supernatant. The band intensities were used to quantify the distribution of Tau protein into pellets versus supernatants (Fig. 6 b, d).

Subject	Age	Gender	Postmortem Interval (h)
Control 1	61	M	16
Control 2	59	M	16.3
Control 3	56	F	17.3
AD 1	79	M	18
AD 2	84	M	11
AD 3	80	M	9.8
PiD 1	73	M	12.4
PiD 2	72	M	4.5
PiD 3	76	F	3.3
PSP 1	71	F	5.3
PSP 2	66	M	8.5
PSP 3	71	M	7

Table S1. Brain tissue used in this study. AD = Alzheimer's disease; PiD = Pick's disease; PSP = progressive supranuclear palsy.

Seeds	Origin	Number of Cells	Number of Puncta
3R Tau Generation 1	AD 1	922	262
	AD 2	944	359
	AD 3	1184	300
4R Tau Generation 1	AD 1	551	181
	AD 2	855	580
	AD 3	1058	744
3R Tau Generation 6	AD 1	1151	373
	AD 2	1540	862
	AD 3	1292	647
4R Tau Generation 6	AD 1	841	369
	AD 2	802	218
	AD 3	573	228
Buffer Control 1		690	7
Buffer Control 2		573	8
Buffer Control 3		747	6

Table S2. Cell and puncta count after seed treatment of HEK293 cells. Overview of the number of cells and puncta counted for replicate experiments shown in Fig. 4 a-c and Fig S14. These data were used to generate the puncta per cell counts presented in Fig. 4 d.
